# Distinct and Predictive Histone Lysine Acetylation Patterns at Promoters, Enhancers, and Gene Bodies

**DOI:** 10.1534/g3.114.013565

**Published:** 2014-08-12

**Authors:** Nisha Rajagopal, Jason Ernst, Pradipta Ray, Jie Wu, Michael Zhang, Manolis Kellis, Bing Ren

**Affiliations:** * Ludwig Institute for Cancer Research, 9500 Gilman Drive, La Jolla, California 92093-0653; † Bioinformatics and Systems Biology Program, University of California, San Diego, La Jolla, California 92037; ‡ Computer Science and Artificial Intelligence Laboratory, Massachusetts Institute of Technology, Cambridge, Massachusetts 02139; § Department of Biological Chemistry, University of California, Los Angeles, Los Angeles, California 90095; ** Department of Molecular and Cell Biology, Center for Systems Biology, The University of Texas at Dallas, Richardson, Texas 75080; †† Cold Spring Harbor Laboratory, 1 Bungtown Rd., Cold Spring Harbor, New York 11724; ‡‡ Department of Applied Mathematics and Statistics, Stony Brook University, Stony Brook, New York 11794; §§ Bioinformatics Division, Center for Synthetic and Systems Biology, Tsinghua National Laboratory for Information Science and Technology, Tsinghua University, Beijing 100084, China; *** Broad Institute of MIT and Harvard, 415 Main Street, Cambridge, Massachusetts 02142; ††† Department of Cellular and Molecular Medicine, Institute of Genomic Medicine, and Moores Cancer Center, University of California, San Diego School of Medicine, La Jolla, California 92093

**Keywords:** histone lysine acetylations, gene bodies, promoters, enhancers, splicing

## Abstract

In eukaryotic cells, histone lysines are frequently acetylated. However, unlike modifications such as methylations, histone acetylation modifications are often considered redundant. As such, the functional roles of distinct histone acetylations are largely unexplored. We previously developed an algorithm RFECS to discover the most informative modifications associated with the classification or prediction of mammalian enhancers. Here, we used this tool to identify the modifications most predictive of promoters, enhancers, and gene bodies. Unexpectedly, we found that histone acetylation alone performs well in distinguishing these unique genomic regions. Further, we found the association of characteristic acetylation patterns with genic regions and association of chromatin state with splicing. Taken together, our work underscores the diverse functional roles of histone acetylation in gene regulation and provides several testable hypotheses to dissect these roles.

In eukaryotes, DNA is packaged into nucleosomes, each consisting of an octamer of histone proteins that can undergo a large number of post-translational modifications (Tan *et al.* 2011). Recent advances in high-throughput technologies such as ChIP-seq have led to the discoveries that various regulatory sequences are characterized by distinct patterns of histone modifications, which have increasingly been used as biochemical signatures for annotation of the genome (Rivera and Ren 2013). For instance, combinations of H3K4me1 and H3K4me3 (Heintzman *et al.* 2007) have been exploited for the identification of enhancers and promoters in mammalian genomes (Won *et al.* 2008; Firpi *et al.* 2010; Fernandez and Miranda-Saavedra 2012; Rajagopal *et al.* 2013). Similarly, combination patterns of H3K4me3 and H3K36me3 were used to uncover a large number of long intergenic noncoding (linc) genes (Guttman *et al.* 2009). Several machine-learning tools have been developed to annotate the histone modification patterns characteristic of various DNA elements (Hon *et al.* 2008; Ernst and Kellis 2012; Rajagopal *et al.* 2013), but given the large number of histone modifications known to exist, there remains a need for more in-depth analysis of histone combination patterns and their relationships to functional sequences.

Histone acetylations are largely considered markers of activity at regulatory elements such as promoters and enhancers, but because of their tendency to co-occur they have been difficult to elucidate the nonredundant roles of these acetylations (Zentner and Henikoff 2013). Histone acetylations are indirectly targeted in the treatment of diseases such as cancer and HIV by the use of HDAC (histone deacetylase) inhibitors (Dinarello *et al.* 2011). Understanding the specific role of histone acetylations at different genomic elements has the potential to improve such therapies by increasing the specificity of targeting. Certain lines of evidence have suggested nonredundant roles of histone acetylation such as the fact that HDACs as well as histone acetyl-transferases (HATs) have unique genomic distributions (Wang *et al.* 2009; Ram *et al.* 2011). A previous study found certain acetylations such as H3K9ac to be present at promoters and H4K16ac along gene bodies (Wang *et al.* 2008). However, the extent to which these acetylations are predictive of particular elements is still unknown.

Although different histone modification patterns have been previously associated with enhancers, promoters, and gene bodies, the discovery of co-transcriptional splicing, the finding that pre-mRNA can be spliced during the process of transcription itself (Listerman *et al.* 2006; Lynch 2006), suggested that histone modification patterns could also be indicative of alternative splicing. Subsequently, it was found that exons are marked by well-positioned nucleosomes and elevated levels of certain methylations, in particular H3K36me3 (Andersson *et al.* 2009; Hon *et al.* 2009; Kolasinska-Zwierz *et al.* 2009; Spies *et al.* 2009). Further supporting this notion, changes in acetylation levels were found to affect alternative splicing (Gunderson *et al.* 2011; Hnilicova *et al.* 2011; Zhou *et al.* 2011). Here, we explore this subject on a genome-wide scale, describing the extent of association of histone modification with alternative splicing in two distinct mammalian cell types.

In a previous study, we developed a random forest-based method (RFECS) that could effectively identify enhancers genome-wide and determine the most informative set of modifications required for this task (Rajagopal *et al.* 2013). Here, we expand the application of this tool to an extended panel of histone acetylation profiles in two distinct mammalian cell types–human embryonic stem cells, ^1^H, and fetal lung fibroblasts, IMR90. Using this approach, we find distinctive patterns of acetylations that are associated with promoters, enhancers, gene bodies, and splice junctions.

## Materials and Methods

### Datasets and processing

All datasets used, including 24 modifications in ^1^H and IMR90, various sequence-specific transcription factors, and DNase-I hypersensitivity sites, were described previously (Rajagopal *et al.* 2013). In addition, the histone modification datasets in H9 were generated by the Ren laboratory and released as part of the Roadmap Epigenome Project and can be accessed using GSE16256. Data normalization for histone modifications, determination of binding sites of transcription factors, training and prediction using RFECS, correlation clustering, and visualization of chromatin patterns are also as previously described (Rajagopal *et al.* 2013).

### Z-score normalization for comparing enhancers and promoters

We pooled equal numbers of distal p300 binding sites and known UCSC TSS overlapping DNase-I hypersensitive sites, representing active enhancers and promoters, respectively. We computed average histone modification levels, measured as input-adjusted RPKM (reads per kilobase per million), between −1 and +1 kb around each of these elements. The Z-score normalized profile for each element was calculated against the mean and SD of the histone modification levels of the entire set of pooled elements. Hence, deviations of the mean z-score profile for the TSS class would be positive for TSS-preferred modifications, whereas it would be negative for p300-preferred modifications. This would be the exact mirror image of the values of the mean z-score values for the p300 class.

### Genome-wide prediction of promoters

To perform supervised prediction of promoters, we created a training set comprising a set of UCSC TSS overlapping DNase-I hypersensitive sites as representative of the active promoter class, and a second class comprising TSS-distal p300 binding sites as well as randomly selected non-p300 regions as background. We used input-adjusted RPKM values of histone modifications (Rajagopal *et al.* 2013) measured in 100-bp bins between −1 and +1 kb around the training set elements as the input features for training this classifier. The RFECS classifier was then used to assign every 100-bp bin in the genome “promoter” or “nonpromoter” class based on a 50% voting percentage, after which promoter peaks were called in a genome-wide fashion as described previously for enhancers (Rajagopal *et al.* 2013). We validated our genome-wide promoter predictions by defining gold standard true positive (TP) and true negative (TN) sets. The former comprises UCSC and Gencode annotated TSS overlapping DNase-I hypersensitivity sites in the particular cell-type, whereas the latter (TN) comprises p300 binding sites, cell-type-specific TFs or DNase-I sites lying within gene desert regions. The TN set was selected to comprise the elements most likely to be mistaken for promoters, due to the enrichment of active modifications. Training and prediction were performed using the RFECS methodology previously applied to prediction of enhancers.

### Computation of variable importance

We used the out-of-bag measure for variable importance (Bylander 2002) to compute importance of either all modifications or just acetylations for various classification or prediction tasks. Because not all modifications had the same replicates, we permuted replicates of each histone modification to create several different combinations and assessed the variable importance for each of these.

### RNA-seq data processing

We first mapped the Illumina-generated mRNA fragments (paired end reads) to the exon trio database TXdb, which we have previously built (Wu *et al.* 2011) using Bowtie version 1 (Langmead *et al.* 2009) for hits with no more than two mismatches. Our sequence mapping is based on the human genome (hg19 assembly, Genome Reference Consortium GRCh37). The fragments are mapped to TXdb to be able to handle transcriptomic variability that arises from alternative splicing. TXdb represents every known contiguous sequence of exons in the human transcriptome as exonic trios and duos, such that mapping to this database allows us to quantify the splicing pattern in terms of the relative abundance of fragments of the different isoforms in this region, locally.

We ran the splicing analysis tool SpliceTrap version 0.90.5 with default parameters, which uses a Bayesian model to estimate inclusion ratios. SpliceTrap uses an inclusion ratio distribution model (estimated from high-confidence data) to reduce noise in the RNA-Seq data without unnecessarily throwing away evidence from real transcriptomic events. Ultimately, it produces inclusion ratio estimates for all splicing events and classifies all local splicing decisions as constitutively spliced exon (CS), alternative donor site (AD), alternative acceptor site (AA), intronic retention (IR), or alternatively spliced exon (CA).

We chose to use SpliceTrap instead of other RNA-Seq analysis tools due to the fact that the SpliceTrap model is exclusively focused on optimizing a local, exon-centric splicing model (which is also our main focus), and that, in our experience, SpliceTrap produces one of the most robust and consistent estimates of inclusion ratios among the tools we compared (Wu *et al.* 2011).

### Splice site usage

We created a measure of splice site usage by using labels associated with each exon–intron boundary to the various categories of splice sites—constitutively spliced exon (CS); alternative donor site (AD); alternative acceptor site (AA); intronic retention (IR); or alternatively spliced exon (CA). Each assignment is accompanied by an inclusion value of the exon with respect to the transcript under consideration. We assigned negative weights to all the cases where inclusion values represent increased inclusion, such as IR, AA (3′ end), AD (5′ end), and we assigned positive weights to the inclusion values that represent decreased inclusion, such as AA (5′ end), AD (3′ end), CA, and CS. The splice site usage value was defined as a weighted mean of the inclusion values, with the weights being the activity of the transcript under consideration. That is, splice site usage for a particular exon–intron boundary is:

$$\text{SS}=-\sum_{j\in A}\sum_{i\in Tj}Incl_{i}*FPKM_{i}+\sum_{j\in B}\sum_{i\in Tj}Incl_{i}*FPKM_{i}$$

i is a particular assignment of an exon with respect to a transcript T_j_

Incl_i_ is the inclusion value of exon–intron boundary in instance i

FPKM_i_ is the RNA-seq FPKM value of the transcript i belonging to set T_j_

A ={IR, AA (3′ end), AD(5′ end)}

B={AA(5′ end), AD(3′ end), CA, CS}

If there was no assignment for any of the seven cases due to weak coverage in that region, then that term was set to 0.

### Identification of chromatin modification patterns at exon–intron boundaries

Using SpliceTrap (Wu *et al.* 2011), we obtained annotations for 286,368 exon–intron boundaries in ^1^H and 246,657 such boundaries in IMR90, of which 232,919 boundaries had annotations in both cell types. In each cell type, we randomly selected a subset of 50,000 sites (∼25%) for unsupervised classification because larger number of sites required many more rounds of selection of the number of clusters to filter out the outliers. We performed fast k-means++ algorithm (Arthur and Vassilvitskii 2007) at the exon–intron boundaries using RPKM-normalized histone modification levels in 100-bp bins between −2 and +2 kb around the boundary as features, and we determined the number of clusters using the minimum value of the Davies-Bouldin measure (Davies 1979). We tested different randomly selected subsets of the data to ensure the results were robust. Further confirmation of the distinctiveness of each of these states was obtained by constructing RFECS classifiers for each cluster against all exon–intron boundaries not assigned to that cluster. We were able to show 100% out-of-bag classification accuracy in ^1^H and more than 95% in IMR90 for each cluster as compared with all others. We used these classifiers to assign all the boundaries that had not been used in the clustering to assign them to the appropriate state.

### Significance calculations for transitions of chromatin state at exon–intron boundaries between ^1^H and IMR90

For computing the significance of the transition from cluster i in ^1^H to cluster j in IMR90, we used a hyper-geometric distribution. Thus, we modeled the probability by using the following analogies to the standard hyper-geometric distribution framework:

total exon–intron boundaries, N = total population
exon–intron boundaries belonging to cluster i in ^1^H, m = elements having desired characteristic
exon–intron boundaries belonging to cluster j in IMR90, n = elements drawn without replacement from the population exon–intron boundaries common to cluster i in ^1^H and cluster j in IMR90, x = number of elements drawn from the total population with the desired characteristic

In Matlab, the p-value of transition from cluster i in ^1^H to cluster j in IMR90 was calculated as:

p-value=1−hygecdf(x,N,n,m)(hypergeometric distribution)

## Results

### Differential histone acetylation patterns at promoters and enhancers

**Figure 1:**
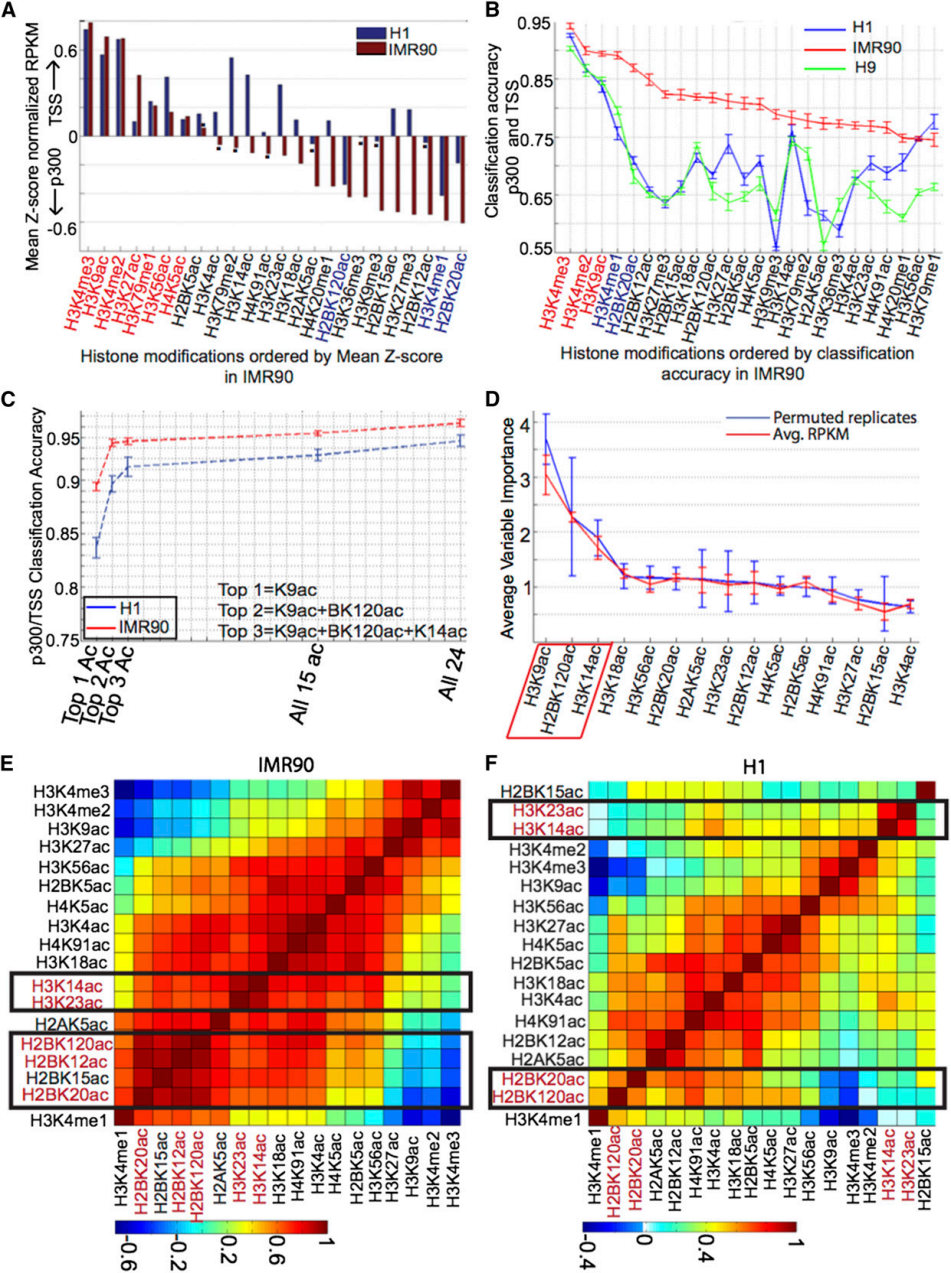
Classification of distal enhancers and promoters. (A) Preference of various histone modifications for either enhancer or promoter using a Z-score normalized score of histone modification levels measured as input-subtracted RPKM (reads per kilobase per million) in ^1^H (blue bars) and IMR90 (red bars).Modifications with preference for promoters, measured as enrichment on the positive y-axis, in both ^1^H and IMR90, are shown indicated in red text color on the x-axis label while preference for enhancers or enrichment on the negative y-axis in both cell-types is indicated in blue text color. (B) Classification accuracy achieved using each of the 24 histone modifications individually to separate enhancers from promoters using RFECS in three distinct cell-lines: ^1^H (blue line), IMR90 (red line), and H9 (green line). H9 is another embryonic stem cell line that was used in this case to see if ^1^H-specific trends were in fact embryonic stem cell–specific. Modifications with the topmost classification accuracy in both ^1^H and IMR90 are shown in either red or blue text color, depending on whether they have preference for promoters or enhancers in both cell types. (C) Comparison of classification accuracy of acetylations with that of all 24 modifications. (D) Ordering of histone acetylations by their out-of-bag variable importance in classification of enhancers against promoters in ^1^H. Correlation clustering of histone acetylations at promoters and enhancers in (E) IMR90 and (F) ^1^H. Acetylations that show similar patterns of co-occurrence in both cell types are indicated in red text color along the axes.

We previously observed that H3K4me1 and H3K4me3 are the most distinctive marks between promoters and enhancers among a limited set of five histone modifications (Heintzman *et al.* 2007). To further define the marks that distinguish these two regulatory elements in genome-wide maps of 24 histone modifications (Rajagopal *et al.* 2013), we compared active TSSs (TSSs overlapping DNase-I HS sites) with an equal number of enhancers defined by TSS-distal p300 binding. After normalization (*Materials and Methods*), we observe that the mean histone modification profile of either class separates clearly into TSS-preferred and enhancer-preferred groups (Figure 1A, positive *vs.* negative axes). We confirmed that the deviation of most of the histone modifications from a set of elements with randomly shuffled labels is statistically significant for total normalized read counts within −1 to +1 kb of the element (Figure 1A; p-value <10^−5^ using Wilcoxon test, except for bars marked by black dots). In both ^1^H and IMR90 cells, we consistently found that H3K4me1, H2BK20ac, and H2BK120ac are significantly enhancer-preferred, whereas H3K4me3, H3K4me2, H3K9ac, H3K56ac, H4K5ac, and H3K27ac are TSS-preferred (Figure 1B). The histone modification profiles in bin sizes of 100 bp between −1 and +1 kb along these elements are also observed to be different from the random set (Supporting Information, Figure S1, A and B, blue *vs.* red).

To assess the importance of each modification in classifying promoters and enhancers, we constructed classifiers using each mark individually. Each classifier was composed of a 20-dimensional vector that was the profile of the histone modification in 100-bp bins between −1 and +1 kb around the element. H3K4me3, followed by H3K4me2 and H3K9ac, showed the highest classification accuracy in both ^1^H and IMR90 (Figure 1B, blue, red). Nearly all modifications showed a classification accuracy of at least 55% (in ^1^H) and 75% (in IMR90), which is above the classification accuracy of 50% expected at chance (we verified that classification accuracy on randomly shuffling labels was found to be ∼50%). Clearly, the most significantly TSS-preferred modifications are H3K4me3, H3K4me2, and H3K9ac. For enhancers, H3K4me1 is the most distinctive, followed by H2BK20ac. In addition, we also observed cell-type-specific contributions. To verify if the modifications specific to ^1^H are due to the distinct biology of stem cells, we repeated our analysis in H9 human embryonic stem cells and observed trends resembling ^1^H (Figure 1B, green *vs.* blue).

We next classified p300 binding sites and TSSs using all 24 marks. Interestingly, H3K4me3 alone achieved the same average accuracy as all 24 modifications in both ^1^H (∼94%) and IMR90 (∼95%) . Next, we examined whether histone acetylation alone could classify these two elements (Figure 1C). The classification accuracy using all 15 acetylations is within 1% of that achieved using all 24 marks. Clearly, acetylations are quite distinctive between the enhancers and promoters.

To identify the specific histone acetylation marks contributing most to the accurate classification of promoters and enhancers, we computed the out-of-bag variable importance (Bylander 2002; Rajagopal *et al.* 2013) for each acetylation. For both ^1^H and IMR90, the top acetylation mark was H3K9ac (Figure 1D, Figure S1C), achieving 85% and 89% classification accuracy, respectively (Figure 1, C and D). The next mark in ordering of variable importance of ^1^H was H2BK120ac, whereas in the case of IMR90 several marks including H2BK20ac shared the same position (Figure 1D, Figure S1C). However, correlation clustering indicates that H2BK20ac and H2BK120ac are highly correlated in both ^1^H and IMR90 (Figure 1, E and F), suggesting that these are redundant modifications. Hence, we selected the top two marks as H3K9ac and H2BK120ac and found that this combination achieved a classification accuracy of within 1% of using all 15 acetylations in IMR90, whereas in ^1^H, this fell short by ∼3%. Including the next mark in the ordering of ^1^H, H3K14ac improved this accuracy by ∼2% (Figure 1C).

In summary, we observed that using acetylation marks alone we could accurately separate these promoters from enhancers nearly as well as using all 24 modifications. Our results indicate differential enrichment of specific acetylations at enhancers and TSS. In particular, H3K9ac, H2BK120ac, and H3K14ac appear to be most informative in combination, of which H2BK120ac is enhancer-preferred whereas the other two are TSS-preferred (Figure 1, A and B).

### Histone acetylation patterns accurately predict enhancers and promoters

Our analysis suggests that histone acetylation patterns are distinct at promoters and enhancers (Figure 1, C and D). Next, we wondered if these acetylations could predict promoters and enhancers genome-wide. As a first step, we applied the RFECS methodology, previously used to predict enhancers (Rajagopal *et al.* 2013), to the prediction of promoters genome-wide (*Materials and Methods*).

**Figure 2:**
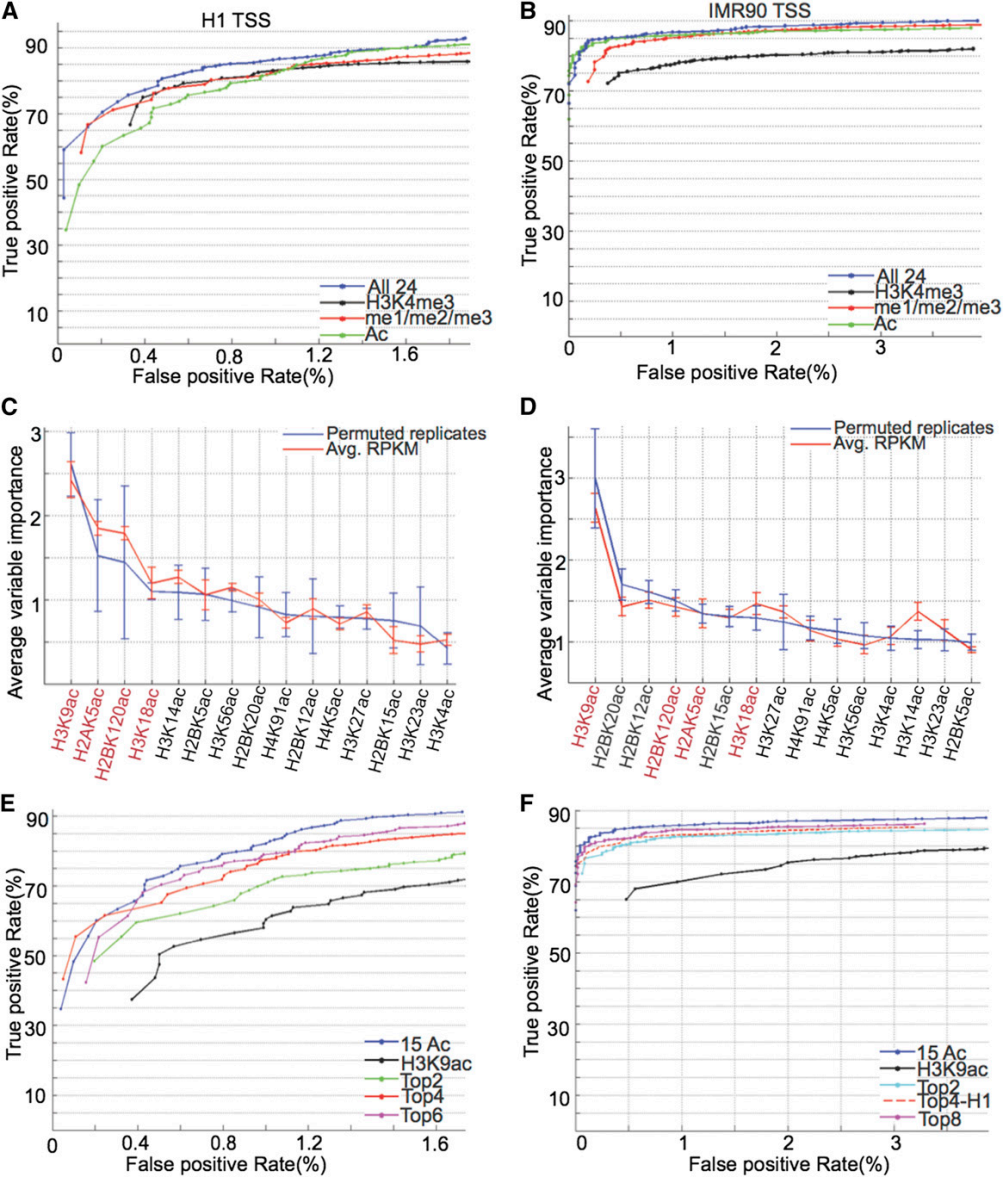
Genome-wide prediction of promoters. Receiver operating characteristic (ROC) curves for prediction of promoters in (A) ^1^H and (B) IMR90 using all 24 modifications (blue), H3K4me3 (black), H3K4me1/2/3 (red), or all 15 acetylations (green). Out-of-bag variable importance for acetylations in making genome-wide prediction of promoters in (C) ^1^H and (D) IMR90. Modification names indicated in red are the ones that show top-most variable importance in both cell types and are considered candidates for selection in the minimal set. ROC curves for prediction of promoters using various minimal combinations of acetylations in (E) ^1^H and (F) IMR90, as compared with the prediction using all 15 acetylations (in blue).

Using all 24 histone modifications, our approach can accurately predict promoters with ∼92% true-positive (TP) rate and ∼1.6% false-positive (FP) rate in ^1^H, whereas in IMR90 we observed even better performance (TP ∼95%, FP ∼0.3%) (Figure 2, A and B). Using the out-of-bag variable measure, we identified H3K4me3 as the most informative mark required to predict promoters, followed by H3K4me2 and H3K4me1 (Figure S2, A and B). In terms of the area under the curve (AUC), this minimal set performs comparably with the set of all 24 modifications in both ^1^H and IMR90 (AUC_min_/AUC_all_ = 0.99) (Figure 2A, red *vs.* blue). While in ^1^H, this set is comparable with using just H3K4me3 (Figure 2A, black *vs.* red); in IMR90, the addition of the two marks leads to an improvement of ∼10% in TP rate as compared with H3K4me3 (Figure 2B, black *vs.* red).

Next, to assess if acetylation can accurately predict promoters, we repeated our analysis on all 15 histone acetylation marks. For IMR90, overall performance was comparable with using all 24 modifications (AUC_ac_/AUC_all_ =0.99) (Figure 2B, green *vs.* blue); for ^1^H, the TP rate was the same for FP rates beyond 1.3% (Figure 2A, green *vs.* blue). To determine which acetylations are the most informative and whether these are robust across cell types, we computed out-of-bag variable importance for acetylations (Figure 2, C and D). H3K9ac is clearly the most informative, whereas the next few marks that are comparable across the two cell types appear to be H2BK120ac, H2AK5ac, and H3K18ac. Several other H2BK-ac also occur among the top ranks in IMR90 (Figure 2D) but are highly correlated with H2BK120ac (Figure 1E).

We then made predictions using just H3K9ac, the top two marks in variable importance for ^1^H and IMR90, and also the predicted most informative set of four acetylations. In ^1^H, there is a significant difference in the ROC (receiver operating characteristic) curve between H3K9ac and the top two marks, H3K9ac and H2BK120ac, and an equivalent increase on including the next two marks, H2AK5ac and H3K18ac (∼8% increase in TP rate for values of FP > 1%) (Figure 2E, black *vs.* green *vs.* red). Even though the performance is not as accurate as using all 15 acetylations, including more marks appears to contribute incrementally to the curves, such as using the top six marks (<2% change in TP for FP > 1%) (Figure 2E, magenta *vs.* red). In IMR90, there is a significant improvement from using the top two modifications as compared with using H3K9ac alone, with difference in TP ranging between 5% and 20% at the same FP (Figure 2F, black *vs.* cyan). Beyond this, improvements appear to be more incremental (<2%), such as in using the predicted minimal set of four modifications (Figure 2F, red dotted) or even on including the top eight marks (Figure 2F, magenta).

Applying the RFECS algorithm (Rajagopal *et al.* 2013) to enhancers, we compared validation and misclassification rates of prediction using just acetylations with that using all 24 marks or the minimal set of H3K4me1, H3K4me2 (or H3K27ac), and H3K4me3. In ^1^H, the validation rate computed based on overlap with known true positives (Rajagopal *et al.* 2013) using just acetylations appears to be comparable to the set of three marks, H3K4me1, H3K4me3, and H3K27ac (Figure S2C), whereas the misclassification rate appears to be within 1% of that using all 24 modifications (Figure S2E). In IMR90, the validation rate using just acetylations is within 3% of that using all 24 modifications (Figure S2D, green *vs.* blue) and a misclassification rate that is within 1% using all 24 modifications (Figure S2F, green *vs.* blue).

Hence, enhancers can also be accurately predicted using just histone acetylation patterns. We computed variable importance for the prediction of genome-wide enhancers using acetylations and discovered H3K9ac, H2BK120/20ac, and H3K14/23ac as the minimal set of acetylations for the prediction of enhancers and confirmed this by comparisons of validation and misclassification rates with performance using all acetylations (data not shown).

In summary, we found acetylations alone to predict genome-wide enhancers as well as promoters quite accurately, indicating that acetylations are not only distinct between the two elements but also predictive. The most informative acetylations in the prediction of promoters were H3K9ac, H2BK120ac, H3K18ac, and H2AK5ac, whereas in the case of enhancers this set was composed of H3K9ac, H2BK120/20ac, and H3K14/23ac.

### Minimal set of modifications to identify active genes

**Figure 3:**
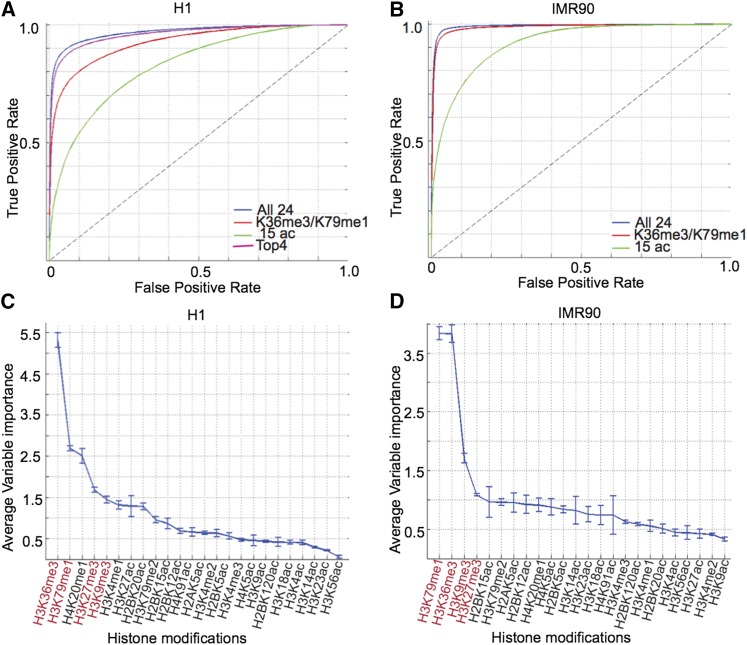
Classification of genic from intergenic regions. ROC curves for classification of genic regions in (A) ^1^H and (B) IMR90 using various combinations of modifications. Out-of-bag variable importance of all modifications in separating genic from intergenic regions in (C) ^1^H and (D) IMR90.

Several histone modifications have been identified as being enriched in the body of active genes (Barski *et al.* 2007). However, the minimum number of modifications required to achieve an accurate prediction of the active gene body is still an unsolved problem. To this end, we identified active RefSeq genes in the ^1^H and IMR90 genomes based on the overlap of their TSS with DNase-I HS sites and RNA-seq above log-value of 2 FPKM. Further, we only considered genic regions lying 2.5 kb away from an annotated TSS. As a true negative set, we identified an equal number of intergenic regions as all those regions not lying within any annotated UCSC, GENCODE, or Refseq gene. We constructed a random forest-based classifier to distinguish these two sets using all 24 histone modifications and observed high sensitivity and specificity at the point of maximum accuracy in both ^1^H (sensitivity = 89.56%, specificity = 94.54%, AUC = 0.97) and IMR90 (sensitivity = 96.34%, 1−specificity = 97.09%, AUC = 0.99) (Figure 3, A and B).

**Figure 4:**
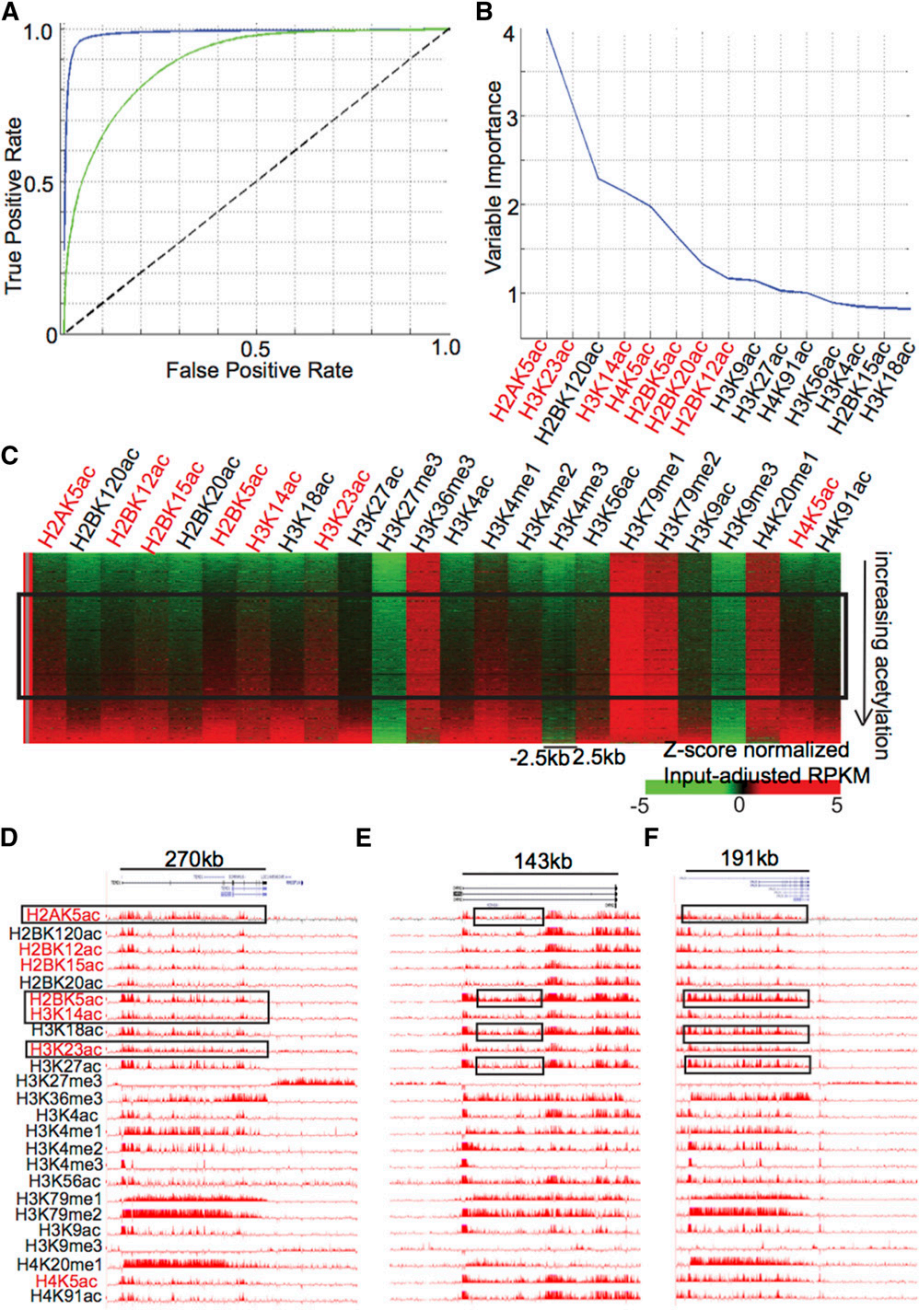
Acetylations within the gene body distal to exon–intron boundaries and DNAse-I hypersensitive sites in IMR90. (A) ROC curves showing classification of distal genic regions using all 24 modifications (blue) or only 15 acetylations (green). (B) Out-of-bag variable importance of acetylations in classification of distal genic regions against intergenic regions. (C) Heatmap showing enrichment of acetylations in genic regions as compared with intergenic ones using a Z-score normalized measure. Only certain acetylations show enrichment in a majority of genic regions as compared with intergenic ones, as indicated by the black box, and emphasized by red text color. These modifications are also shown in red in (B) and can be seen to be among the top-most marks for variable importance in separation of genic from intergenic regions. UCSC genome browser snapshot of genes (D) TEAD1, (E) CHRM2, and (F) CALD1, showing enrichment of acetylations as compared with neighboring intergenic regions.

In both ^1^H and IMR90, the top two informative marks are H3K36me3 and H3K79me1, which rank well above all other marks (Figure 3, C and D). By AUC analysis, the performance of these two marks alone is equivalent to that of all 24 marks in IMR90 (AUC_K36me3,K79me1_/AUC_all_ = 100%), although it is somewhat lower in ^1^H (AUC_K36me3,K79me1_/AUC_all_ = 96%) (Figure 3, A and B, green). We found that the two marks ranked next that were common to both cell types were H3K27me3 and H3K9me3 (Figure 3, C and D). These modifications may be important because of their relative depletion in genic regions and enrichment in larger intergenic regions (Figure 4D). By including these marks, our classifier achieved almost the same accuracy as all 24 marks in ^1^H (^1^H: AUC_top 4_/AUC_all_ = 99%) (Figure 3A, magenta *vs.* blue). Thus, we conclude that the minimal set of modifications required to predict genes within 1% accuracy of the set of all modifications is between 2 and 4, with H3K36me3 and H3K79me1 being the most informative modifications.

### Acetylations at the gene body

Next, to assess if gene body acetylation can distinguish genic from nongenic regions, we constructed a supervised classifier using only histone acetylations. Supporting this notion, acetylations show an ROC curve that is well above the line of no discrimination in both ^1^H and IMR90 (Figure 3, A and B). However, the performance of acetylations is lower (^1^H:AUC_ac_/AUC_all_ = 0.85,IMR90: AUC_ac_/AUC_all_ =0.92) than that achieved using all 24 marks or even the top four nonacetylation marks (Figure 3, A and B, green *vs.* blue). For instance, in IMR90, the sensitivity and specificity are 81.24% and 84.94%, respectively, as compared with 95.27% and 97.5% for all 24 marks, at default parameters.

Given the lower proportion of genic regions predicted with acetylations, we asked if this was because of the lower fractions of gene bodies recovered by acetylations or the existence of distinct categories of genes that are either completely acetylated or not, defined based on their predictability using just acetylations. To this end, we examined the distribution of fractions of genes recovered by either case and that using all 24 marks leads to 90–100% recovery of most genes, whereas the fractions recovered by just acetylations appear to be more evenly distributed (Figure S3, A and B). The partial recovery of certain genes using acetylations may indicate a bias toward certain elements within the gene. Because previous studies have found associations of acetylations with the splicing of certain genes (Gunderson *et al.* 2011), we tested the hypothesis that acetylations might have a preference for exonic regions or exon–intron boundaries and found this to be true in both ^1^H and IMR90 (File S1, Figure S3).

Although acetylations clearly show a bias toward exonic boundaries, a sizeable fraction of genes (12.7% in ^1^H; 16.11% in IMR90) that can be recovered up to >90% using acetylations alone still exists (Figure S3, A and B). Distal regulatory elements lying within intronic regions are enriched in acetylations. Because we wanted to see if the gene bodies have a distinct acetylation pattern independent of such intronic enhancers, we selected only those genic regions that are at least 2.5 kb away from a known DNase-I HS or an exon–intron boundary. Now, we calculated the classification rate of these filtered genic *vs.* intergenic regions using all 24 modifications and just acetylations (Figure 4A, Figure S4A). It can be seen that the recovery using just acetylations is still well above the line of no discrimination (significance stats), with a maximum classification accuracy of ∼70% in ^1^H and ∼80% in IMR90 (Figure S4A, Figure 4A).

Because gene body acetylations appeared to be quite discriminative in the case of IMR90, we further examined which acetylations are most enriched within the gene body. H2AK5ac, H3K23ac, H3K14ac, H4K5ac, and H2BK5ac were found to be among the top acetylations in order of variable importance (Figure 4B), and also showed enrichment in a majority of genic regions on normalization to intergenic background (Figure 4C). We selected long genes, such as TEAD1 (Figure 4D),CHRM2 (Figure 4E), and CALD1 (Figure 4F), that could be classified to more than 90% against an intergenic background. It can be seen that several modifications such as H2AK5ac, H3K14ac, H3K23ac, and H2BK5ac seem to cover a large proportion of the gene as compared with the neighboring intergenic region. Although some of this may be accounted for by the presence of punctate regulatory elements, there are also regions that show diffuse enrichment of the aforementioned acetylations, emphasized in Figure 4E in the black boxes.

In ^1^H, similar analysis yielded a different set of acetylations that were seen to be among the most enriched at gene bodies, with H3K27ac being the top-most in terms of variable importance (Figure S4B). On visualizing the enrichment of various histone modifications at genic regions *vs.* intergenic ones, it does appear that H3K27ac has a ubiquitous but low presence (Figure S4C). The enrichment of several acetylations within the gene body can also be at the active gene PTPRJ, which is in sharp contrast to a neighboring intergenic block with H3K9me3 enrichment (Figure S4D).

Finally, we examined if acetylations have any functional significance in gene bodies. Gene expression levels were slightly higher at acetylated genes (Figure S4, E and F), showing a low but significant Pearson correlation coefficient of 0.2 in ^1^H and 0.14 in IMR90. Further, we examined if the genes with higher acetylation had specific associations with functional annotations. In ^1^H as well as IMR90, mRNA processing and RNA binding were among the significantly enriched terms (Table 1). In addition, each cell-type showed different categories that were enriched such as that of genes involved in regulation of intracellular protein transport in IMR90 (Table S2) or genes involved in mRNA splicing in ^1^H (Table S1).

**Table 1:** GO terms for acetylation-rich genes in ^1^H and IMR90

GO Term	Description	^1^H p-value	IMR90 p-value
GO:0006397	mRNA processing	5.90E−09	7.19E−04
GO:0010467	gene expression	4.79E−05	4.79E−05
GO:0003723	RNA binding	3.21E−04	1.03E−05

### Histone modification signatures at exon–intron boundaries

Previous observations of co-transcriptional splicing suggest that specific chromatin signatures may be associated with splicing (Kolasinska-Zwierz *et al.* 2009). As a preliminary investigation, we chose to analyze the predictive power of the histone modifications under study in predicting exon–intron boundaries from the genic background. Using histone modification profiles (in 100-bp bins) between −2 and +2 kb around the exon–intron boundaries, we were able to classify all known boundaries from genic background with an accuracy of 87% in ^1^H (AUC_all_ = 0.94) or 85.5% in IMR90 (AUC_all_ = 0.93). We then investigated the contribution of each histone modification under study to the prediction. On computing variable importance for each of the histone modifications with respect to the aforementioned classification, we found H3K36me3 followed by H3K79me1 to be the most informative and H3K36me3 alone could classify the boundaries within 3% of the accuracy achieved using all 24 modifications (AUC_k36me3_/AUC_all_ ∼96%).

To further investigate the association of histone modifications at exon–introns with function, we identified various splicing events from paired-end RNA-seq in both ^1^H and IMR90 (Xie *et al.* 2013) using SpliceTrap (Wu *et al.* 2011). The algorithm classified each local splicing decision as being one of constitutively spliced exon (CS), alternative donor site (AD), alternative acceptor site (AA), intronic retention (IR), or alternatively spliced exon (CA) with respect to its flanking exons. Based on the diversity of isoforms of a particular gene, this can cause one exon to be part of multiple alternative splicing events. In each such splicing event, we may characterize the splicing decision in terms of the inclusion ratio, defined as the ratio of quantified expression level of the inclusion isoform divided by the sum of quantified expression levels of both inclusion and exon-skipped isoforms. Further, each exon can also be quantified in terms of the exonic activity measured as FPKM (fragments per kilobase per million mapped reads). We aim to use these two quantifications at the exonic level to tease out correlations between histone modification signals and splicing activity.

**Figure 5:**
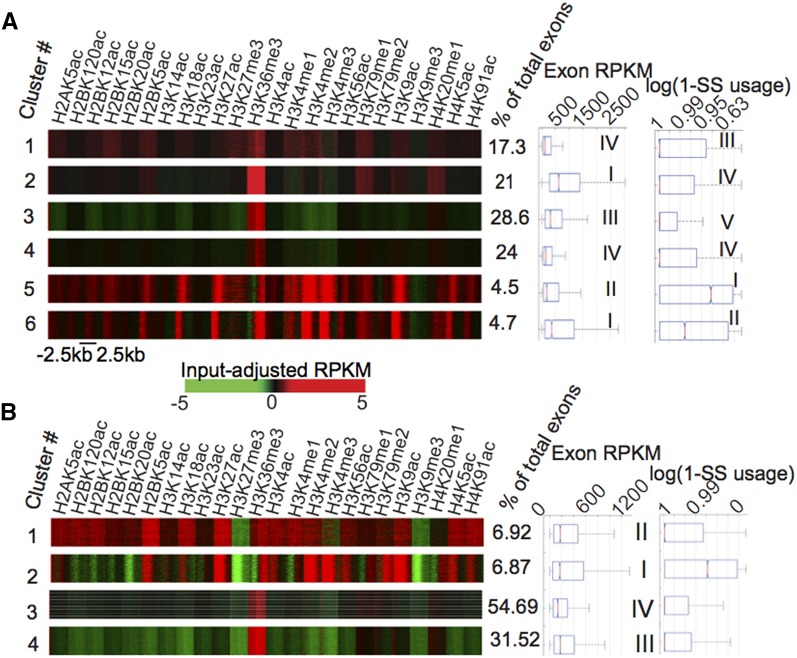
Chromatin modification patterns at exon–intron junctions in ^1^H and IMR90. (A) Six distinct chromatin modification patterns at exon–intron junctions with corresponding levels of exonic activity (panel 2) and splice site retention (panel 3). (B) Four distinct chromatin modification patterns at exon–intron junctions with corresponding levels of exonic activity (panel 2) and splice site retention (panel 3). Ranks associated with each of these parameters are shown for the clusters, in ^1^H as well as in IMR90, based on significant differences in either exonic activity or splice site retention between the clusters, measured using a Wilcoxon test.

Because there is a wide diversity of splicing activity in the transcriptome, the multiple signals associated with an exon–intron boundary may lead to the observation of a convoluted histone modification signal. As a first step toward deconvoluting such putative chromatin modification signals, we discovered all possible chromatin modification patterns at exon–intron junctions using a fast k-means++ algorithm (Arthur and Vassilvitskii 2007) (see *Materials and Methods*). Six distinct clusters are observed in ^1^H (Figure 5A), with varying levels of acetylations as well as other gene body marks such as H3K36me3, H3K79me1, and H4K20me1. Each of these clusters were characterized in terms of their distinctiveness from the genic background by classifying the exons assigned to the cluster against the genic background using either all 24 modifications or just acetylations (File S1, Figure S5, A and C). Overall, state 2 is unclassifiable against background using just acetylations, indicating that the weak acetylation signature is comparable with the gene body, whereas other states were found to be either overenriched (states 1, 5, 6) or underenriched (states 3, 4) for acetylations as compared with the rest of the gene (File S1, Figure S5, A and C). It is worth noting that only those states with enrichment of acetylations appear to have presence of H3K79me1 as well.

In IMR90, however, we observed four distinct chromatin modification patterns (Figure 5B). In common with ^1^H, there is an “enhancer-like” cluster, cluster 1 (cluster 1 in ^1^H), and “promoter-like” cluster, cluster 2 (cluster 5 and 6, ^1^H), based on enrichment of H3K4me1 and me3, respectively. As in ^1^H, these two are significantly enriched in acetylations with respect to genic background, whereas state 4 is significantly depleted (File S1, Figure S5, B and D).

The learned histone modification states in ^1^H cells are ranked in decreasing order of exonic activity based on calculations of statistical significance of the difference of mean RNA-seq FPKM (fragments per kilobase per million) levels between clusters using a Student’s *t*-test (Figure 5A, panel 2). In ^1^H, there appears to be a positive correlation with the level of H3K36me3, which is apparent as clusters 2 > 3 > 4 that show significantly decreasing trends of activity also have correspondingly decreasing H3K36me3 (spearman correlation for clusters 1 to 4 = 0.59; p-value < 2.2×10^−308^). However, “TSS”-like signatures (clusters 5 and 6) appear to be even more highly active, irrespective of H3K36me3 enrichment. The same trend may be observed in IMR90, where cluster 3 with the lowest enrichment of H3K36me3 also has the lowest activity (spearman correlation for clusters 1, 3, and 4 = 0.47; p-value < 2.2×10^−308^), and “TSS-like” state 2 has the maximum exonic activity (Figure 5B, panel 2).

In summary, H3K36me3 can accurately classify most exon–intron junctions from genic background. We identified multiple distinct chromatin states at both ^1^H and IMR90 that are associated with varying levels of exonic activity. We found that there was considerable variation in the levels of acetylations at exon–intron boundaries, many of which were either highly enriched or highly depleted in acetylations with respect to the rest of the gene.

### Chromatin modification patterns are predictive of splice-site usage

As described in the section above, an exon can be part of multiple different splicing events such as constitutively spliced exon (CS), alternative donor site (AD), alternative acceptor site (AA), intronic retention (IR), or alternatively spliced exon (CA) with respect to its flanking exons. A single exon–intron junction can have multiple assignments of inclusion values based on the transcript under consideration. Hence, we further developed a metric to characterize the overall splice site usage for every exon–intron boundary based on an expression-weighted average of its inclusion ratio in all transcripts (*Materials and Methods*).

Chromatin modification clusters are ranked in decreasing order of retention or increasing order of splice site usage in ^1^H using a Wilcoxon test with a p-value cutoff of 10^−5^ (Figure 5A, panel 3). A clear trend is observed where the greater the enrichment of acetylations, the stronger the tendency for retention, with clusters 6, 5, and 1 having the maximum tendency for retention (Figure 5A, panel 3). In IMR90 as well, the highly acetylated clusters 2 and 1 showed significantly higher retention of the boundary (Figure 5B, panel 3, ranked I and II based on a p-value cutoff of 10^−5^).

**Figure 6:**
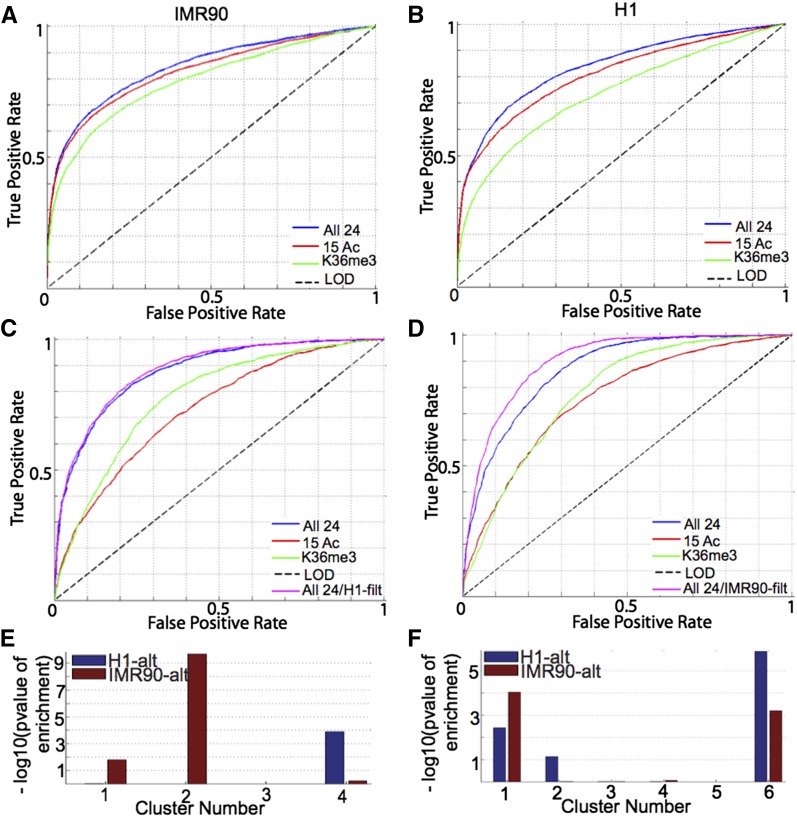
Associations of chromatin modification patterns with splicing. (A–D) ROC curves for the classification of alternatively spliced exon–intron junctions against constitutively spliced ones using all 24 modifications (blue), 15 acetylations (red), or H3K36me3 (green) for classification of (A, B) group I exon–intron junctions comprising intronic retention (IR), alternative 5′ end usage (AD), and alternative 3′ end usage (AA) in (A) IMR90 and (B) ^1^H. Group II exon–intron junctions comprising alternatively spliced exon (CA) in (C) IMR90 and (D) ^1^H. Negative logarithm of the p-value of enrichment of alternatively spliced exons exclusive to ^1^H (blue) or IMR90 (red) in (E) IMR90 and (F) ^1^H.

We asked to what extent we could predict retention of exon–intron junctions based on chromatin modifications as input features. We defined the constitutive class of exon–intron boundaries as those that have the maximum possible value of inclusion ratio, 0.999, in all transcripts of which they are a part. We defined two categories of alternatively spliced exon–intron boundaries based on their contribution to splice-site usage: group I class of boundaries comprising IR, AD (5′ end), and AA (3′ end) contribute negatively to splice-site usage, whereas group II class of boundaries comprising CA contributes positively to splice-site usage, as defined above. Using all 24 modifications, we obtained a maximal classification accuracy of ∼70% and AUC of 0.75 for group I exon–intron boundaries in IMR90 (Figure S6B, black). Although this is clearly greater than expected at random, we asked if we could further improve the classification accuracy by taking into consideration other factors. For instance, exon–intron boundaries within close proximity of each other may share the same chromatin signature, which would cause difficulty in classification. To verify this, we filtered any retained exon–intron boundary within different distances of the constitutive exon–intron boundaries and found a steady improvement in accuracy of classification with filtering distance (Figure S6B, black to red). Now, if we consider filtering the group I elements for any constitutive exon–intron boundaries, we actually observed a worsening of the performance (Figure S6B, black *vs.* dotted blue). We obtained the best possible accuracy of classification with an AUC of 0.84 and maximal accuracy of 77.1% by using a filtering distance of 10 kb for determining the set of distal constitutive exon–intron boundaries in IMR90 (Figure 6A, blue). In ^1^H, we observed the same trend (data not shown) and obtained a maximal accuracy of 76.5% and AUC of 0.84 for classification of group I exon–intron junctions against distal constitutive ones (Figure 6B, blue).

Histone lysine acetylations had been observed to be enriched at clusters with greater degree of retention (Figure 5, A and B). To further explore the relative importance of histone lysine acetylations, we classified the group I exon–intron junctions against the distal constitutive ones and obtained a comparable classification accuracy as using all 24 modifications (Figure 6, A and B, blue *vs.* red, ^1^H: AUC_ac_/AUC_all_ = 0.96, IMR90: AUC_ac_/AUC_all_ = 0.98). Previous studies had shown H3K36me3 to be distinctive between alternatively spliced exons and constitutively spliced ones (Hon *et al.* 2009). As compared with acetylations, H3K36me3 was able to achieve a much lower accuracy of classification (Figure 6, A and B, blue *vs.* red, ^1^H: AUC_k36_/AUC_all_ = 0.88, IMR90: AUC_k36_/AUC_all_ = 0.94), indicating the stronger association of group I alternatively spliced exons with acetylation signatures, rather than H3K36me3.

On classification of group II alternatively spliced exons against a constitutive background, we achieved a maximal accuracy of ∼66% and AUC of 0.71 in IMR90 (Figure S6B, black). We considered the case of classifying distal alternative *vs.* constitutive ones and found a steady improvement of classification accuracy on increasing filtering distance for removing nonretained exon–intron boundaries in the vicinity of the group II alternative class (Figure S6B, solid blue to red). At best, we achieved a maximal accuracy of 80% and AUC of 0.88 for classifying distal group II alternative exon–intron boundaries against the constitutive background in IMR90 (Figure 6C, blue). In ^1^H, we achieved a similarly high level of accuracy of performance with a maximal accuracy of 78% and AUC of 0.87 (Figure 6D, blue). In the case of group II junctions, H3K36me3 was more effective than histone lysine acetylations in classifying alternative boundaries against constitutive ones (Figure 6, C and D, green *vs.* red), although neither acetylations nor H3K36me3 could achieve comparable performance as that using 24 modifications (^1^H: AUC_k36_/AUC_all_ = 0.9, IMR90: AUC_k36_/AUC_all_ = 0.89).

Patterns in both cell types were also associated with specific splice variants to see if there were significant associations with these (Figure S6, C and D). Alternative donor sites or 5′ splice sites were enriched in the promoter-like clusters in both cell types as compared with any other state. However, surprisingly, all other splice variants also have a greater tendency to occur proximal to such promoter-like signatures. PLEKH3 is a gene that is predicted by SpliceTrap to have a series of retained exon–intron boundaries in ^1^H that are constitutively spliced in IMR90. This gene can be seen to have chromatin state changes associated with alternative splicing and retention (Figure S7A). The reverse can be seen in the gene VIM where certain exons that are constitutive in ^1^H are retained in IMR90 (Figure S7B). In both cases, the set of exons undergoing various types of retention, excluding alternative 5′ site usage, are indicated by a black box and can be seen to be covered by the expansion of H3K4me3 signal in the cell type with alternate usage. Another observation to note was that state 4 in ^1^H appeared to be preferential for exons with both ends constitutively spliced, whereas states 1, 5, and 6 show preference for other events such as alternative acceptor sites or intronic retention (Figure 5A, Figure S6D).

In conclusion, using chromatin modification information, we were able to achieve accuracy as high as 80% for the classification of alternatively spliced exon–intron junctions from a constitutively spliced background. We observed improvement in classification accuracy on considering a constitutive background distal to any retained exon in case of group I exons and by considering an alternative class distal to constitutive exons in case of group II exons. This suggests the effect of proximal chromatin signature on neighboring exons. Retained exon–intron boundaries are highly enriched for histone lysine acetylations, especially intronic retention, alternative 3′ end usage, and alternative 5′ end usage. “Enhancer-like” and “promoter-like” chromatin states that appear to be associated with splice site retention are common to both cell types, of which the latter is the most strongly associated with a variety of splice site variants, not just alternative 5′ sites.

### Dynamics of chromatin modification states at splice sites

Certain chromatin modification clusters in ^1^H appear to be analogous to ones in IMR90 based on the patterns of modifications, such as the “enhancer-like” state 1 (^1^H) with state 1 (IMR90), and the “promoter-like” state 5 and state 6 (^1^H) with state 2 (IMR90) (Figure 5, A and B). However, the other clusters are not so easily comparable in terms of chromatin modifications. In this regard, we examined if particular states in ^1^H have a tendency to correspond to ones in IMR90 based on the number of exon–intron junctions that are common to the states in the two cell types. We computed the p-value of transitions between the six states in ^1^H to the four states in IMR90 using a hyper-geometric distribution (*Materials and Methods*) and significant transitions, based on a p-value < 2.2×10^−308^, are enumerated in Table 2. It appears that the chromatin state transitions are in keeping with the overall ranking in terms of splice site usage. For instance, state 2 in ^1^H and state 4 in IMR90 show significant transitions even though their chromatin modification patterns do not appear to be the same. However, both these clusters are ranked immediately after the “promoter-like” and “enhancer-like” states in terms of their splice site usage. Such a trend is in keeping with the fact that the change in splice site usage across the two cell types is relatively small. For instance, if we assume any exon junction with splice site usage <0.9 to be called alternative, then only 1.92% of the total exons undergo any change at all in their splice site usage between ^1^H and IMR90.

**Table 2:** Significant chromatin state transitions at exon–intron junctions between ^1^H and IMR90

Cluster	IMR90 Cluster 1	IMR90 Cluster 2	IMR90 Cluster 3	IMR90 Cluster 4
^1^H cluster 1	Yes	No	No	Yes
^1^H cluster 2	No	No	No	Yes
^1^H cluster 3	No	No	Yes	No
^1^H cluster 4	Yes	No	Yes	No
^1^H cluster 5	Yes	Yes	No	No
^1^H cluster 6	No	Yes	No	No

We observed that we could obtain a considerably higher accuracy of classification of group II alternatively spliced exons in ^1^H if we considered a negative set that was composed of constitutive exons in both ^1^H and IMR90, rather than just ^1^H with an improvement in maximal accuracy of approximately 4% (Figure 6D, magenta *vs.* blue). However, there is not much difference in accuracy of classification on using this constitutive background in IMR90 (Figure 6C, magenta *vs.* blue). This suggests that certain constitutive exons in ^1^H may be “pre-marked” for alternative splicing in IMR90. To validate this, we created two sets of junctions based on splice site usage—one that is alternatively spliced in ^1^H but not IMR90 and another that is alternatively spliced in IMR90 but not ^1^H (Figure 6F,blue *vs.* red). Both the acetylation-rich clusters 1 and 6 in ^1^H (Figure 5A) are significantly enriched for cell-type-specific retained junctions whether it is in ^1^H or IMR90 (Figure 6F). However, in IMR90, the corresponding acetylation-rich clusters 1 and 2 are not significantly enriched for ^1^H-exclusive retention events (Figure 6E). Hence, it may be that the states in ^1^H are pre-marked for alternative splicing in IMR90 because they are undifferentiated cells that contain the tendency for alternative splicing in future differentiated cells as well. Because IMR90 is a fully differentiated cell type, it does not show similar tendencies.

Overall, it appears that only a small proportion (<2%) of exons undergo alternative splicing changes between ^1^H and IMR90. The chromatin modification patterns at exon–intron boundaries changes across ^1^H and IMR90 in such a manner to correspond to the splice site usage corresponding to the cluster, rather than the actual enrichment of various modifications. Also, constitutive exon–intron boundaries in ^1^H may be pre-marked by an alternative splice site signature for use in later differentiated cell types such as IMR90.

## Discussion

Chromatin modifications distinguishing promoters and enhancers have previously been identified as H3K4me1 and H3K4me3 (Heintzman *et al.* 2007). Besides these two, we find that several additional histone modifications, especially histone acetylations, can also reliably distinguish these regulatory elements. In particular, H3K9ac, H3K23ac, and H3K14ac are promoter-preferred, whereas H2BK120ac and H2BK20ac are enhancer-preferred. Overall, histone acetylation is not only distinctive between the two regulatory elements but also informative enough to predict promoters and enhancers genome-wide. These observations potentially lead to several hypotheses regarding differences in mechanisms of functioning of these two regulatory elements. H2BK120 has been shown to have a ubiquitination modification that is present at active promoters and exclusive of H2BK120ac (Gatta *et al.* 2011). This exclusivity may explain the presence of H2BK120ac at enhancers, and may suggest the lack of H2BK120Ub at these elements. Understanding the dynamics of the H2BK120 acetylase, KAT3 (Gatta *et al.* 2011), and the H2BK120 ubiquitin ligase, RNF20 (Hwang *et al.* 2003; Zhu *et al.* 2005), may lead to further understanding of differences between enhancers and promoters.

In addition to enhancers and promoters, acetylations were found to be quite informative in delineating gene bodies. It was previously observed that certain acetylations showed elevated levels at the promoter region as well as the transcribed regions of active genes in CD4^+^ T-cells (Wang *et al.* 2008). We wanted to investigate if specific patterns of acetylations were distinctive of the gene body alone by investigating the predictive power of these acetylations in ^1^H and IMR90 cell types. We found extensive enrichment of H2AK5ac, H2BK120ac, H3K14ac, and H3K23ac along gene bodies, and acetylations alone can achieve 80% accuracy in predicting gene bodies. Some studies have shown PCAF to be regulating H3K14ac (Lau *et al.* 2000), also known to be part of an elongation-competent form of RNA-polymerase II (Cho *et al.* 1998). This factor may be involved in the maintenance of gene body acetylations in IMR90. Tip60 and HDAC6 have also been characterized as being within gene bodies (Wang *et al.* 2009), the former of which is known to acetylate H2AK5 (Jeong *et al.* 2011). Hence, given the patterns of acetylations within gene bodies and prediction of genes enriched in these, there is a potential to generate hypotheses regarding the combinatorial localization of HATs and HDACs within specific genes.

Acetylations within the gene body are especially enriched near exon–intron junctions of retained exons. We described two groups of such exon–intron junctions—one that comprised events contributing to negative splice site usage such as intronic retention (IR), alternative 3′ end (AA), and alternative 5′ end (AD) usage, and another that contributed to positive splice site usage comprising alternatively spliced exons (CA). We found that both these groups showed significant association with proximal chromatin state but had differential associations with histone lysine acetylations. Histone lysine acetylations were found to be highly discriminative in classifying group I exons against a set of distal constitutive exon. However, H3K36me3 depletion appeared to be more distinctive of the class of group II exons. We observed improvement in classification accuracy on considering a constitutive background distal to any retained exon in case of group I exons and by considering an alternative class distal to constitutive exons in case of group II exons. Possibly, the chromatin signature of these group I exons may be more permissive for allowing constitutive splicing in neighboring exons, whereas the chromatin signature of group II exons may not be as permissive for allowing constitutive splicing and may be more strictly restricted to distal group II exons. In case of constitutive and alternative exons within close proximity to each other, factors other than the chromatin state may play an important role in regulating the splicing. One of these could be the effect of distal regulatory elements interacting with the splice-site junctions (Mercer *et al.* 2013). The role of such elements in splicing can be further studied using a chromosomal conformation captures technique such as 4C (Zhao *et al.* 2006). It was also observed that many acetylation-rich, constitutive exons in ^1^H are alternatively spliced in IMR90. Such a hypothesis may be further tested by including detailed splicing and chromatin formation across many human cell lines, both from early and late lineages.

Hence, we observed patterns of histone acetylations that are specific to promoters, enhancers, and genic regions. Such observations are in keeping with many previous studies regarding the localization of chromatin modifiers at these elements and suggest further testable hypotheses regarding the combinatorial enrichment of potential chromatin modifiers at these regions, which could lead to a better understanding of the mechanism of functioning of enhancers, promoters, and genes.

## Supplementary Material

Supporting Information
