# Supplementary material for: Distinct and Predictive Histone Lysine Acetylation Patterns at Promoters, Enhancers, and Gene Bodies
Source: G3 (Bethesda). 2014 Aug 12;4(11):2051–63. doi: 10.1534/g3.114.013565 (PMC4232531; doi:10.1534/g3.114.013565)
Supplement: Supporting Information [file supp_4_11_2051__index.html]

Distinct and Predictive Histone Lysine Acetylation Patterns at Promoters, Enhancers and Gene Bodies — Distinct and Predictive Histone Lysine Acetylation Patterns at Promoters, Enhancers, and Gene Bodies — Supporting Information 

# Distinct and Predictive Histone Lysine Acetylation Patterns at Promoters, Enhancers, and Gene Bodies

## Supporting Information for Rajagopal *et al.*, 2014

**Files in this Data Supplement:**

- Supporting Information - Figures S1-S7, File S1, and Table S1 (PDF, 4.3 MB)
- Figure S1 - Differential histone modifications between enhancers and promoters. (PDF, 251 KB)
- Figure S2 - Genome-wide prediction of promoters and enhancers (PDF, 442 KB)
- Figure S3 - Recovery of genic regions using acetylations (PDF, 192 KB)
- Figure S4 - Acetylations within the gene body distal to exon-intron boundaries and DNAse-I hypersensitive sites in H1 (PDF, 1.2 MB)
- Figure S5 - Enrichment of acetylations at exon-intron boundaries for each chromatin state (Fig.5A,B) with respect to genic background (PDF, 1.1 MB)
- Figure S6 - Association of chromatin modification patterns with splice-site usage (PDF, 1.1 MB)
- Figure S7 - "Promoter-like" chromatin states are associated with various splice variants (PDF, 976 KB)
- File S1 - Fractional Acetylation of gene bodies (PDF, 90 KB)
- Table S1 - GO terms for acetylation-enriched genes in H1 (.xls, 276 KB)
