# Supplementary material for: Distinct and Predictive Histone Lysine Acetylation Patterns at Promoters, Enhancers, and Gene Bodies
Source: G3 (Bethesda). 2014 Aug 12;4(11):2051–63. doi: 10.1534/g3.114.013565 (PMC4232531; doi:10.1534/g3.114.013565)

SUPPLEMENTARY MATERIAL

Fractional Acetylation of gene bodies

In order to understand the distribution of acetylations within genic regions, we defined two categories- 100bp bins that are accurately classified as genic using just acetylations and those that are accurately classified using all 24 marks but not acetylations, both at the same false positive rate. Overall, it was found that, 99% of all the active refseq genes in IMR90 and 96.54% in H1 could be atleast partially recovered using acetylations, which is comparable to that achieved using all 24 modifications. However, when we looked at the distribution of fractions of genes recovered by either case, it was found that all 24 marks leads to 90-100% recovery of most genes, while the fractions recovered by just acetylations appear to be more evenly distributed (Supplementary Figure 3A,B).

In order to examine the bias of acetylations towards exon-intron boundaries, we compared the proportion of acetylated bins recovered within exonic regions to the proportion recovered without acetylations, and found this difference to be highly significant in both H1 and IMR90 (p-value<2.2X10^-308, hyper-geometric). For those acetylated bins that were recovered in intronic regions, we calculated the distance of recovered bins to the exon-intron boundary and observed a significant bias towards acetylated bins being nearer to the exon-intron boundary (Wilcoxon test, p-value<2.2X10^-308) for both H1 and IMR90 (Supplementary Figure 3C,D).

Enrichment of histone acetylations at exon-intron boundaries within learned chromatin modification clusters

Six distinct chromatin modification patterns were discovered at exon-intron boundaries in H1(Fig.5A). We built a classifier for exon-intron junctions within each of these clusters against a genic background, and computed the classification rate for out-of-bag values(Breiman 2001). In Supplementary Figure 5A, it can be observed that all clusters except for state 4 show over 80% classification accuracy against genic background. Unlike all modifications, classification with acetylations yielded a maximum accuracy >85% for all states except state 4, which was unclassifiable against genic background(~50% classification accuracy). This suggests the weak acetylation pattern at state 4 maybe akin to the acetylation enrichment within the gene body. To verify this, we normalized histone acetylation levels (measured as reads per kilobase per million, RPKM in 100bp bins) within -2.5 to +2.5 kb around exon-intron junctions against genic background using a Z-score (Supplementary Figure 5C). In Supplementary figure 5C, it can be seen that histone acetylation levels for state 4(black) are close to zero, as expected, while clusters 1,2,5 and 6 are enriched for acetylations (above zero line) and cluster 3 is depleted for acetylations(below zero line). These enrichments and depletions can be considered as significant since classification accuracy expected at random would be 50% and for all of these chromatin states, the classification accuracy is over 85% using just acetylations (Supplementary Figure 5A).

In IMR90, we obtained 4 distinct chromatin modification patterns (Fig.5B). All of the clusters show maximum classification accuracy of >85% using all modifications and >70% using just acetylations (Supplementary Figure 5B). Hence, it would appear that all of these exon-intron junctions are either enriched or depleted in acetylations with respect to the gene body. In order to investigate this, we look at the Z-score normalized profile of histone modifications (Supplementary Figure 5D) as in H1. Cluster 1 and cluster 2 appear to be enriched with respect to genic background, while cluster 3 and cluster 4 appear to be depleted.

Hence, in both H1 and IMR90, we observe categories of exon-intron boundaries with varying degrees of enrichment or depletion of histone acetylations with respect to other genic regions.

SUPPLEMENTARY TABLES

Table S1. GO terms for acetylation-enriched genes in H1

Table S2.GO terms for acetylation-enriched genes in IMR90

Available online at http://enhancer.ucsd.edu/nisha/ac_paper_supp_tables

SUPPLEMENTARY FIGURE LEGENDS

Figure S1.Differential histone modifications between enhancers and promoters.

A,B.) Preference of various histone modifications from -1kb to +1kb around an enhancer or promoter using a Z-score normalized score (blue) as compared to the randomly shuffled class labels (red) in A.) H1 B.) IMR90 C.) Ordering of histone acetylations by their out-of-bag variable importance in classification of enhancers against promoters in IMR90.

Figure S2. Genome-wide prediction of promoters and enhancers.

A,B) Ordering of all 24 histone modifications by their out-of-bag variable importance for the prediction of promoters in A.)H1 and B.)IMR90. C,D)Validation rates and E,F) misclassification rates for enhancer predictions at various voting percentage cutoffs in C,E.)H1 and D,F.) IMR90 using all 24 modifications(blue), H3K4me1/2/3 (red), H3K4me1,H3K4me3 and H3K27ac(cyan) and 15 acetylations(green).

Figure S3. Recovery of genic regions using acetylations

A,B.) Fraction of gene body predicted in A.)H1 and B.)IMR90 using all 24 modifications(blue) or just acetylations(red). C,D.) Fraction of predicted 100bp bins lying at various distances from exon-intron boundaries using all 24 modifications(blue) or just acetylations(red) in C.)H1 and D.)IMR90. E,F.) Distance of predicted 100bp bins from exon-intron boundaries versus activity of exons in E.)H1 and F.)IMR90.

Figure S4. Acetylations within the gene body distal to exon-intron boundaries and DNAse-I hypersensitive sites in H1

A.) ROC curves showing classification of such distal genic regions using all 24 modifications(blue) or only 15 acteylations(green).B.) Variable Importance of acetylations in classification of distal genic regions C.)Heatmap showing enrichment of acetylations in genic regions as compared to intergenic ones using a Z-score normalized measure. D.)UCSC genome browser snapshot of gene PTPRJ showing enrichment of acetylations as compared to neighbouring intergenic region. E,F.) Gene expression levels versus fractional enrichment of acetylations within the gene body in E.)H1 and F.)IMR90.

Figure S5. Enrichment of acetylations at exon-intron boundaries for each chromatin state (Fig.5A,B) with respect to genic background

A,B.) Maximum classification accuracy of each state against genic background using all 24 modifications(blue) or 15 acetylations (red) in A.) H1 and B.) IMR90. C,D.) Histone acetylation RPKM levels at exon-intron boundaries Z-score normalized against levels at genic regions, distal to DNAse-I HS and exon intron boundaries, in C.) H1 and .D) IMR90.

Figure S6. Association of chromatin modification patterns with splice-site usage

A,B) ROC curves showing classification of A.) Group I class of exon intron junctions or B.) Group II exon intron junctions against constitutive background, at various distances for filtering

either constitutive exons neighbouring alternative ones (indicated by CSXkb in the legend) or at distances for filtering the alternative exons for the ones proximal to constitutive exons (indicated by Altxkb) . Filtering distance parameter is increased in increments of 2.5 kb from 0 to 10kb.C.) Association of various types of splicing events with chromatin states in IMR90(Fig.5B). D.) Association of various types of splicing events with chromatin states in H1(Fig.5A).

Figure S7. "Promoter-like" chromatin states are associated with various splice variants. Snapshots from UCSC genome browser of A.) PLEKH3 showing alternative splicing of several exons in H1 as compared to IMR90 B.) VIM showing altternative splicing of several exons in IMR90 as compared to H1.

SUPPLEMENTARY REFERENCES

Breiman, L. 2001. Random Forests. *Machine Learning* **45:** 5-32.

Supplementary Figure 1

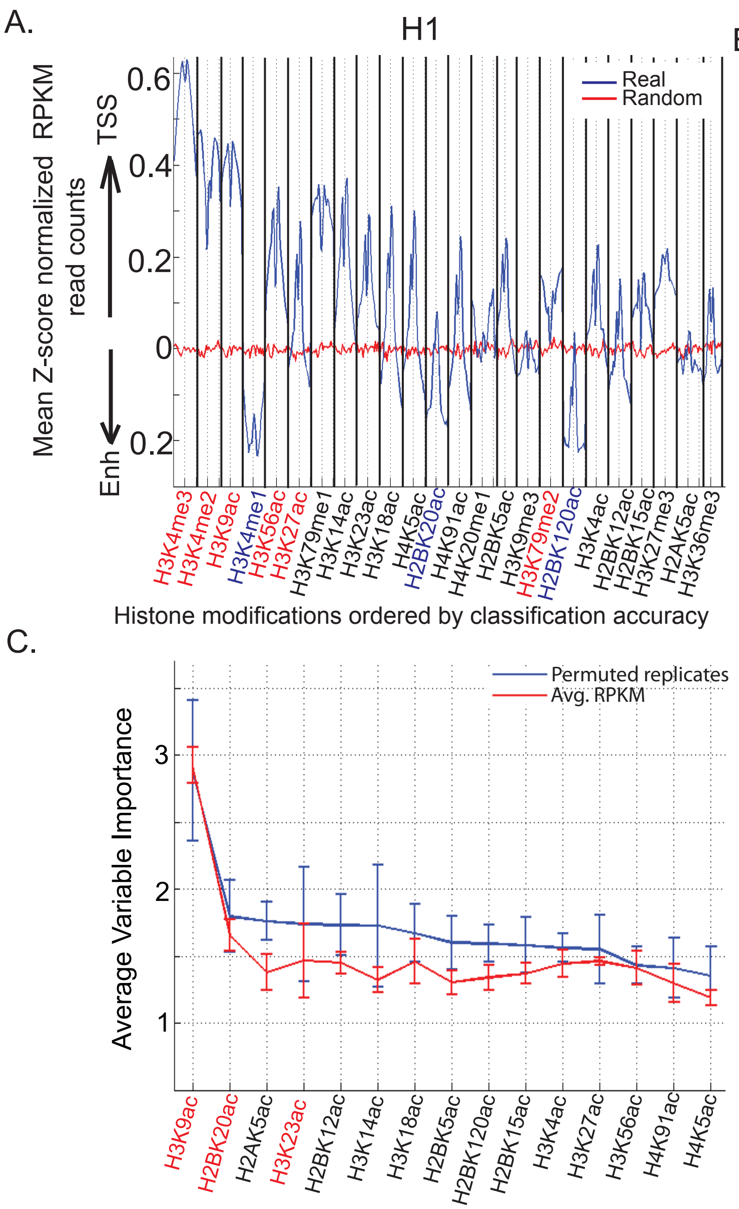

Supplementary Figure 2

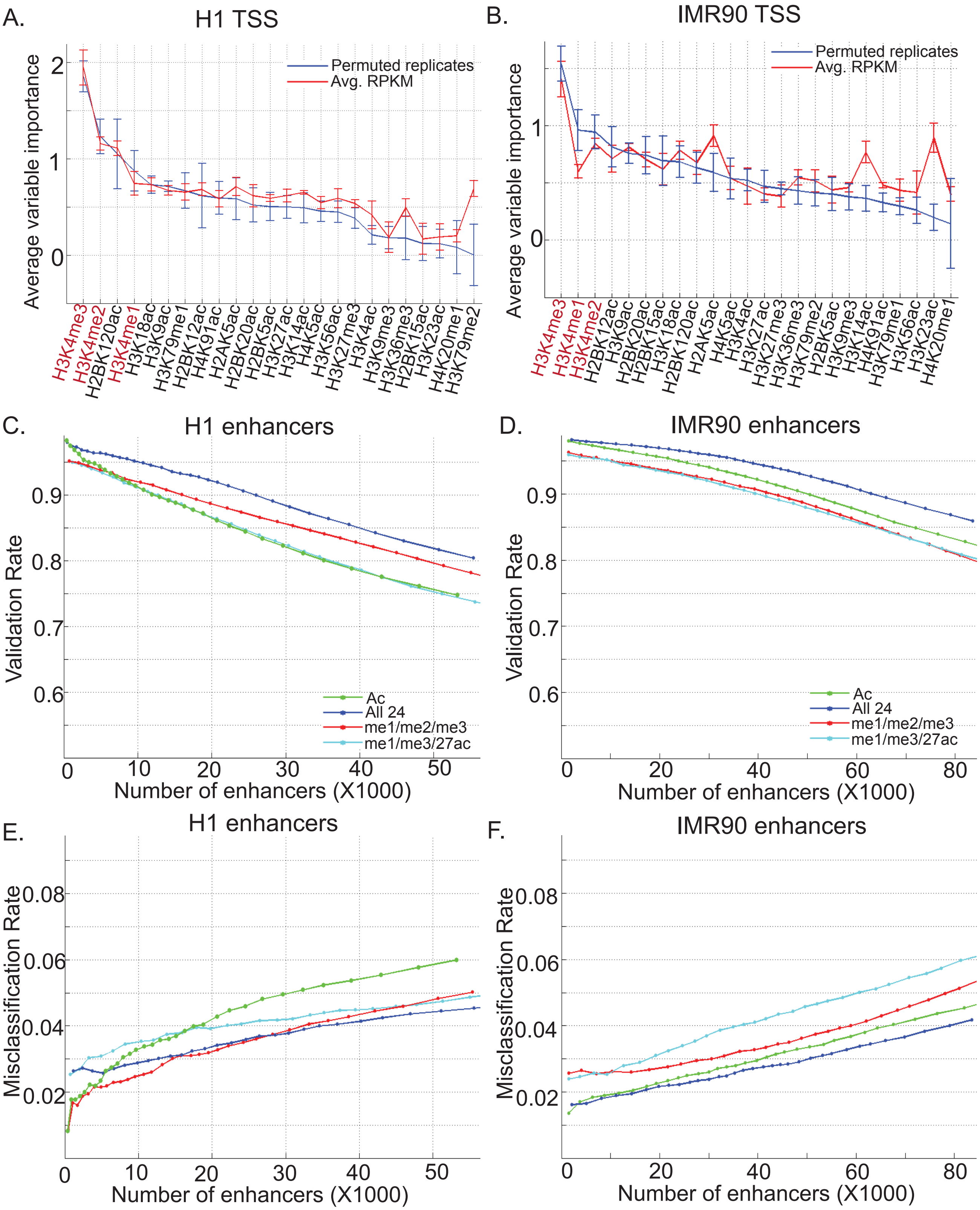

Supplementary Figure 3

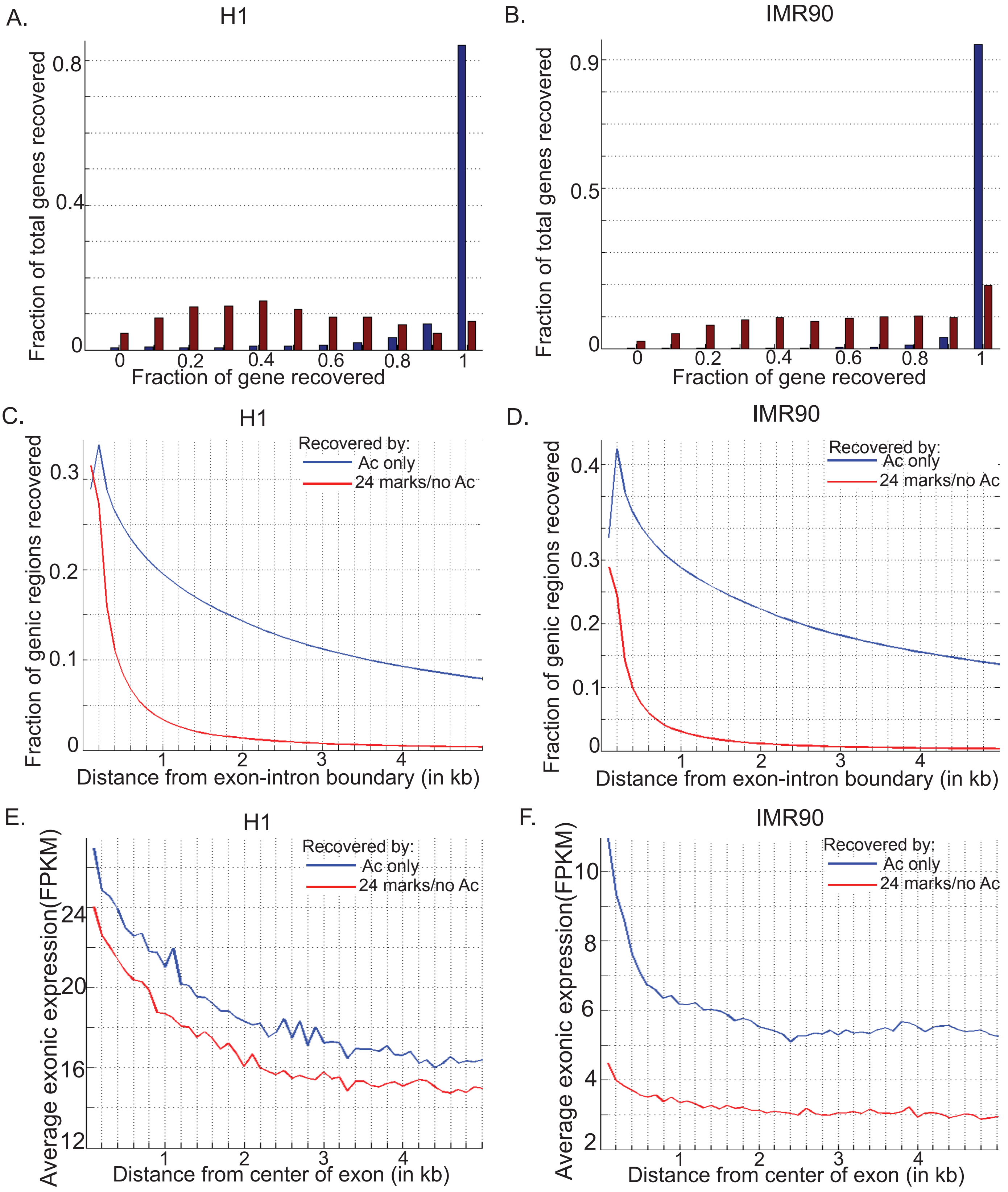

Supplementary Figure 4

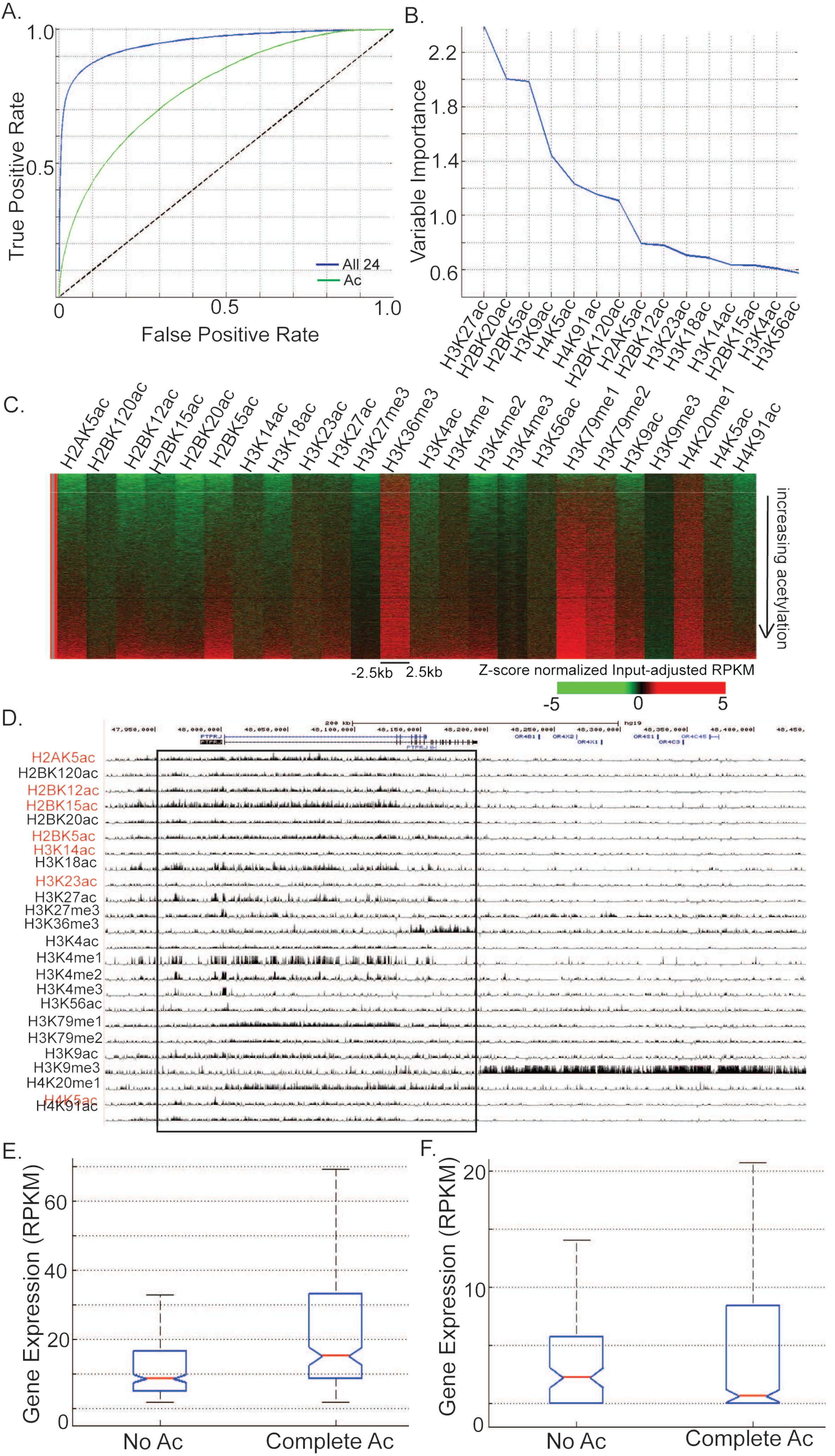

Supplementary Figure 5

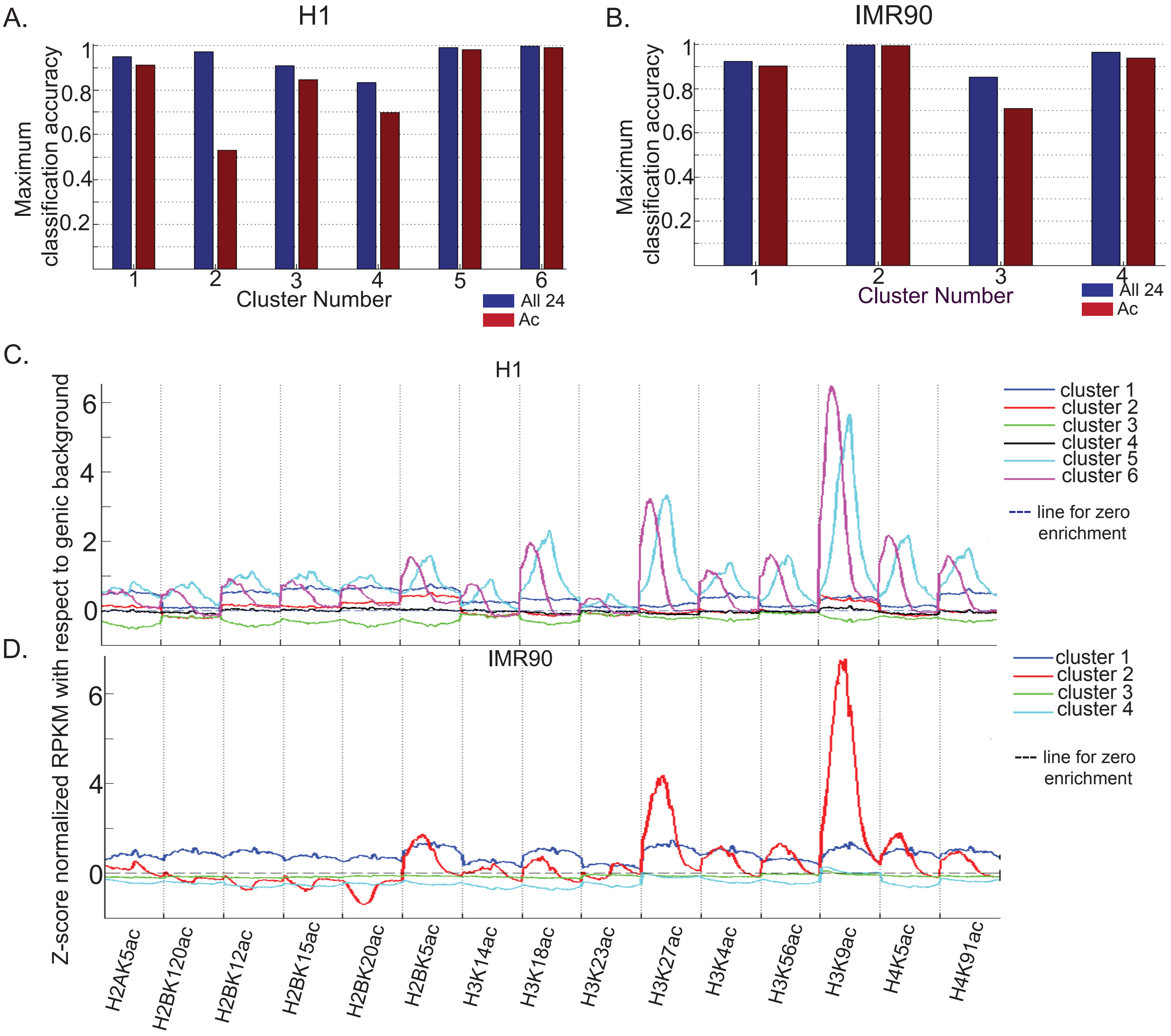

Supplementary Figure 6

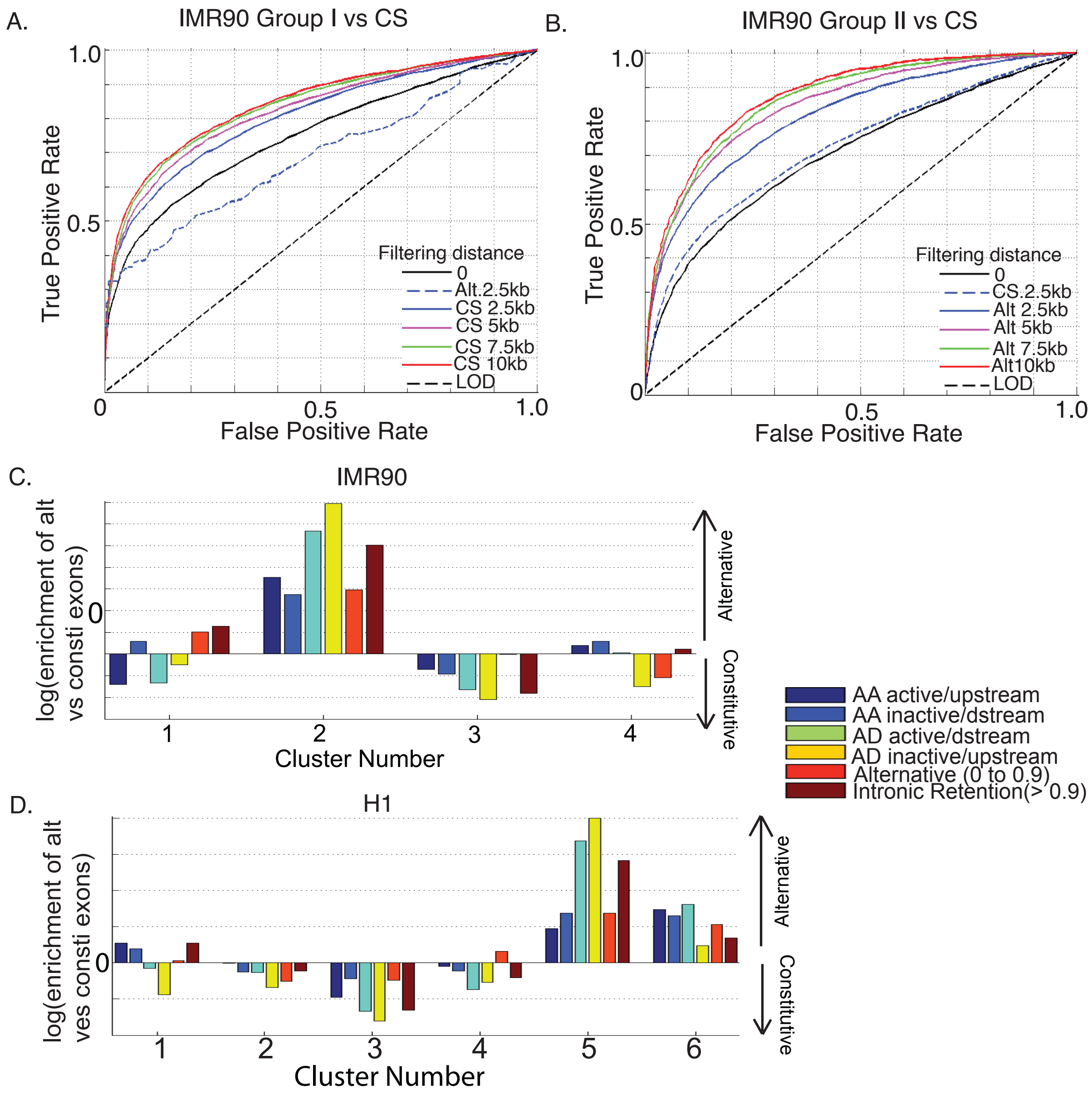

Supplementary Figure 7

A.

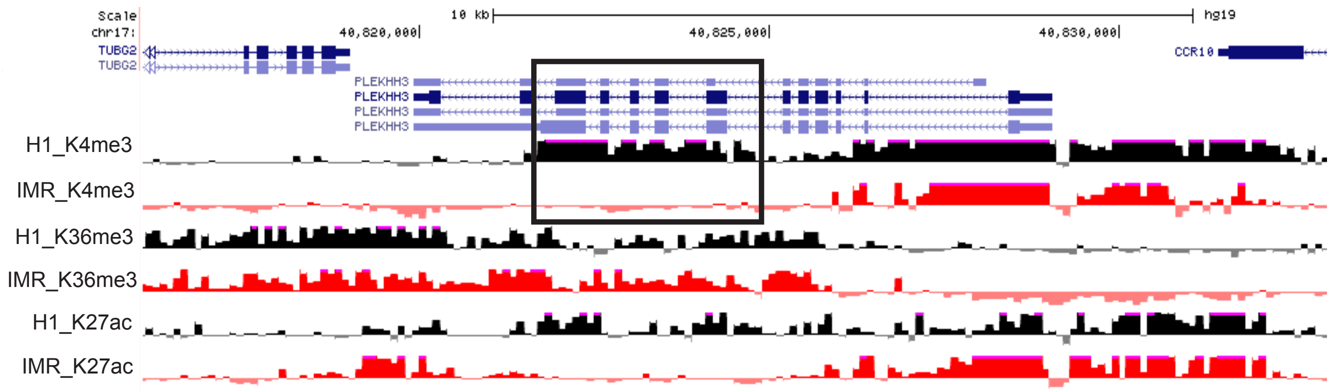

B.

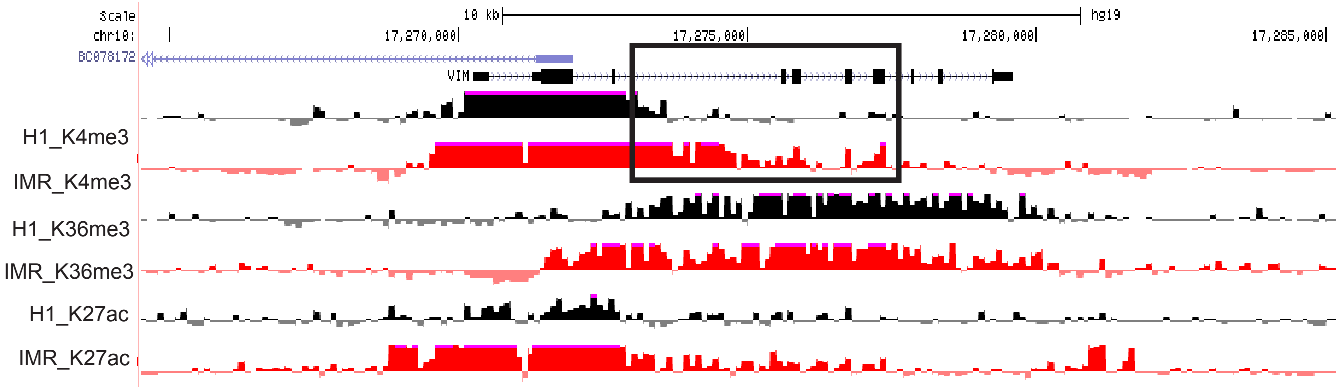

Supplement: Supporting Information [file supp_g3.114.013565_013565SI.pdf]
