# Supplementary figures and images for: Distinct and Predictive Histone Lysine Acetylation Patterns at Promoters, Enhancers, and Gene Bodies
Source: G3 (Bethesda). 2014 Aug 12;4(11):2051–63. doi: 10.1534/g3.114.013565 (PMC4232531; doi:10.1534/g3.114.013565)

Supplementary Figure 1

A.

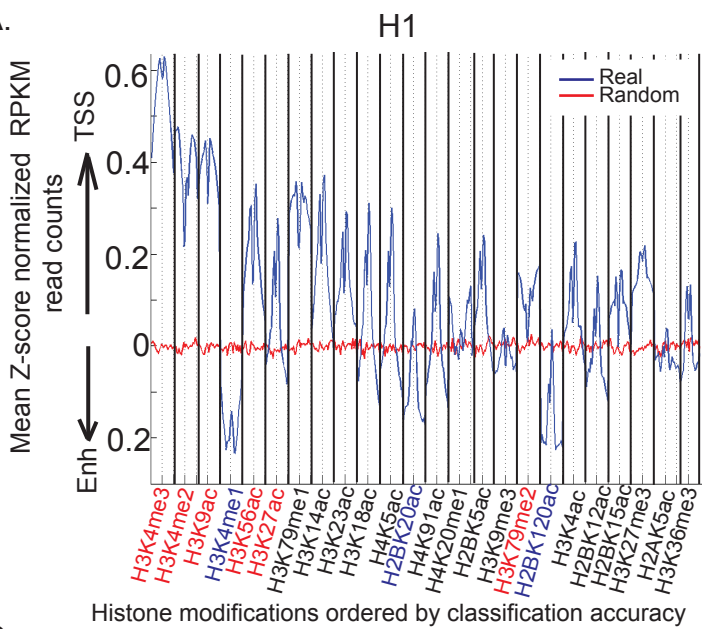

B.

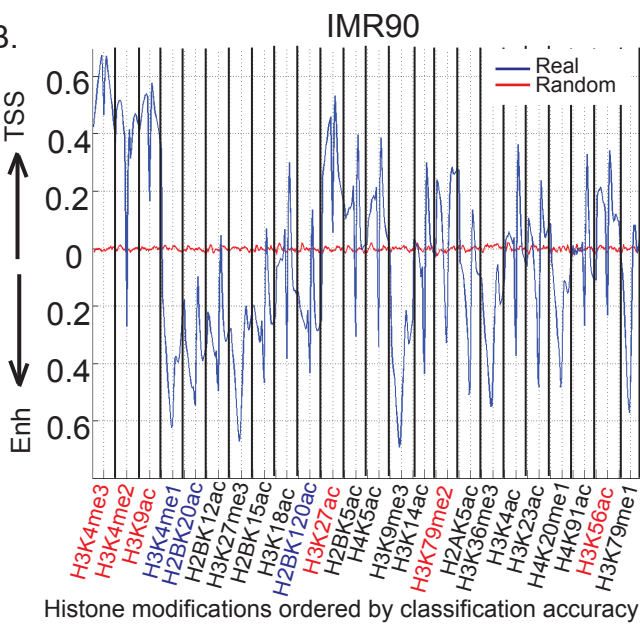

C.

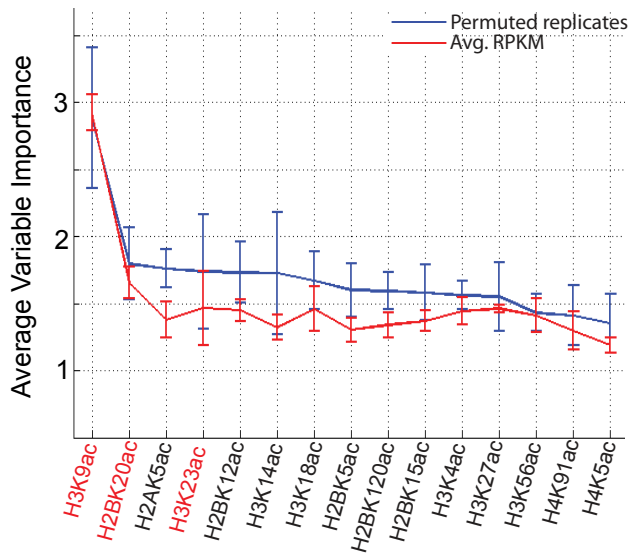

Supplement: Supporting Information [file supp_g3.114.013565_FigureS1.pdf]

Supplementary Figure 2

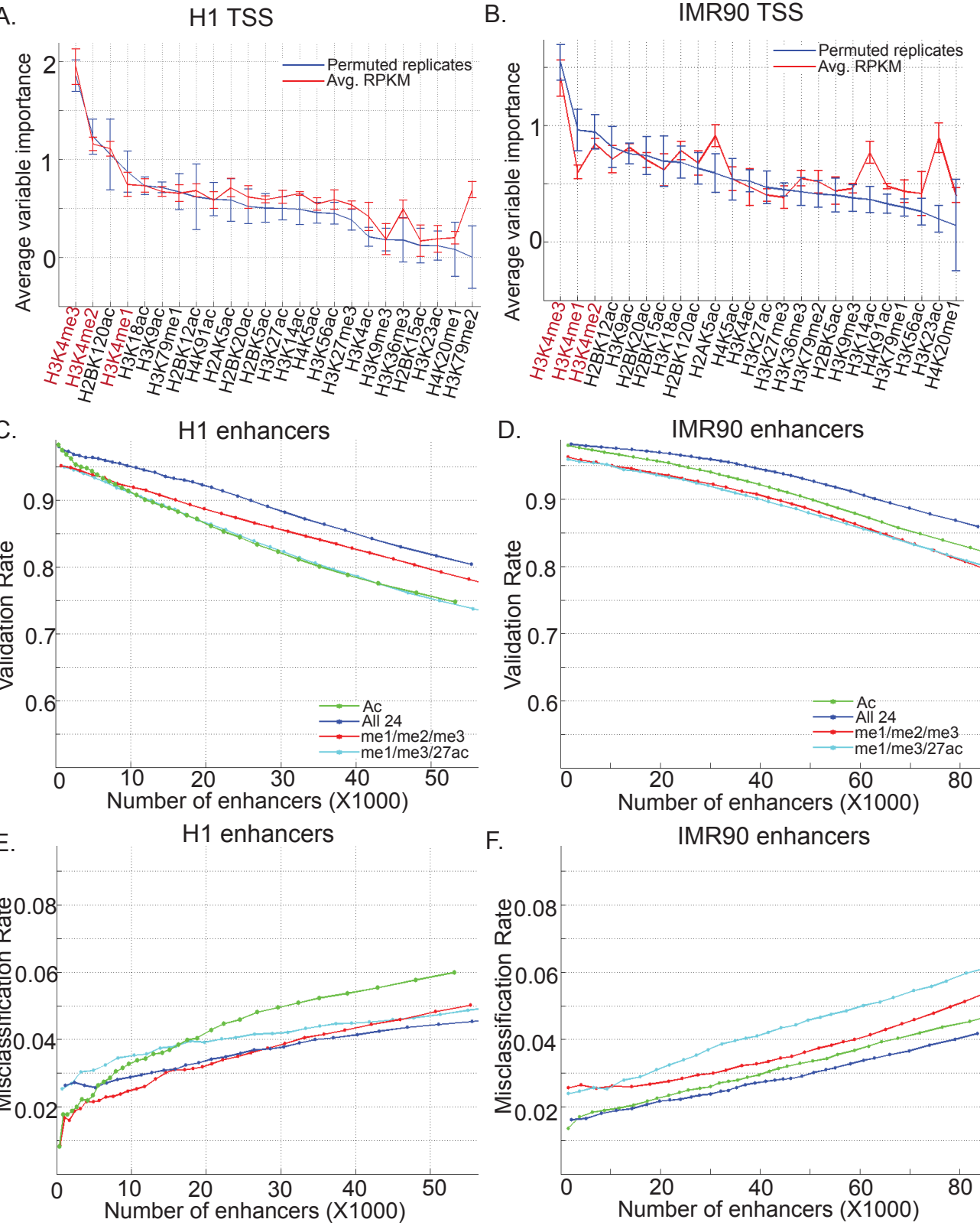

Supplement: Supporting Information [file supp_g3.114.013565_FigureS2.pdf]

Supplementary Figure 3

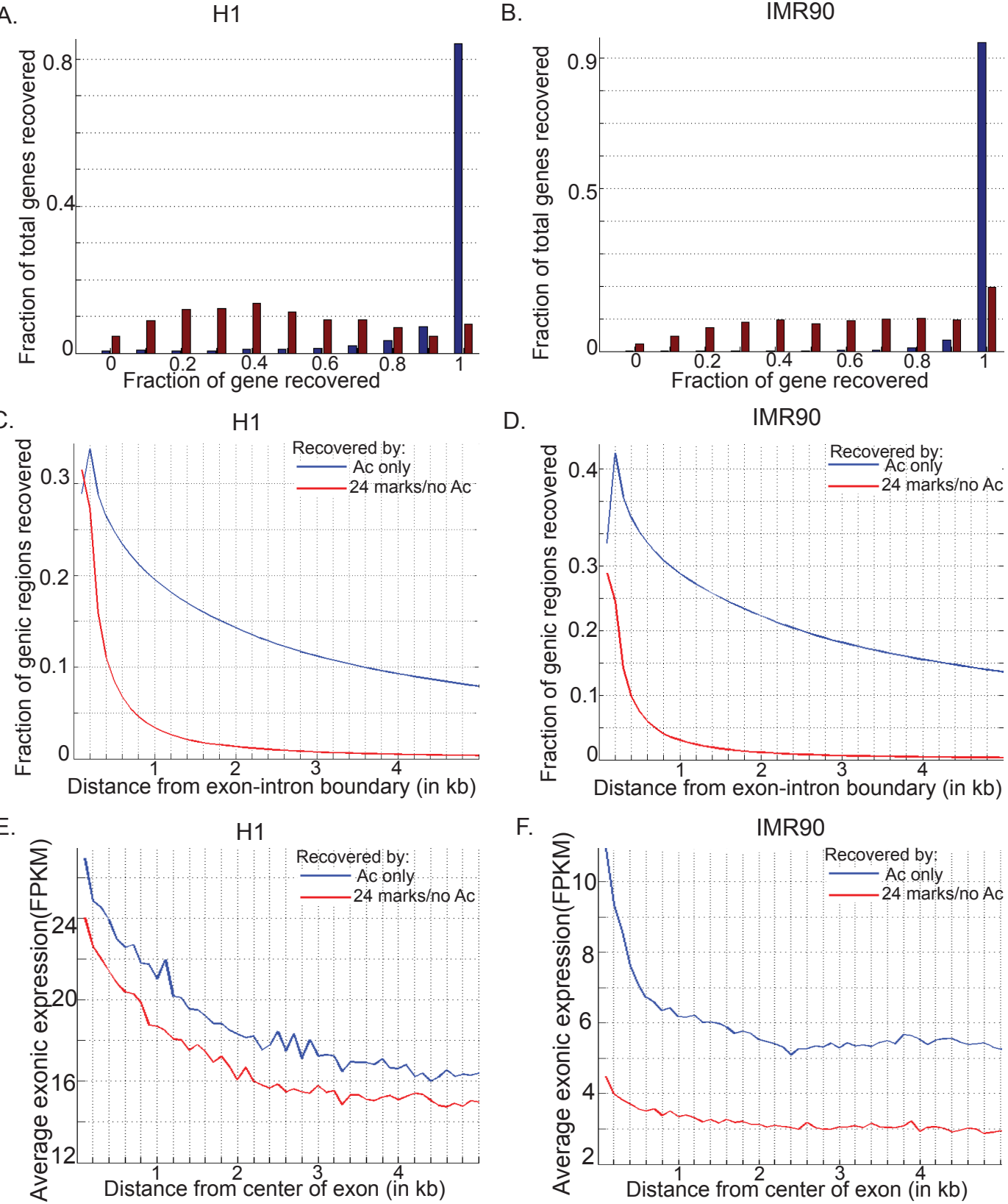

Supplement: Supporting Information [file supp_g3.114.013565_FigureS3.pdf]

Supplementary Figure 4

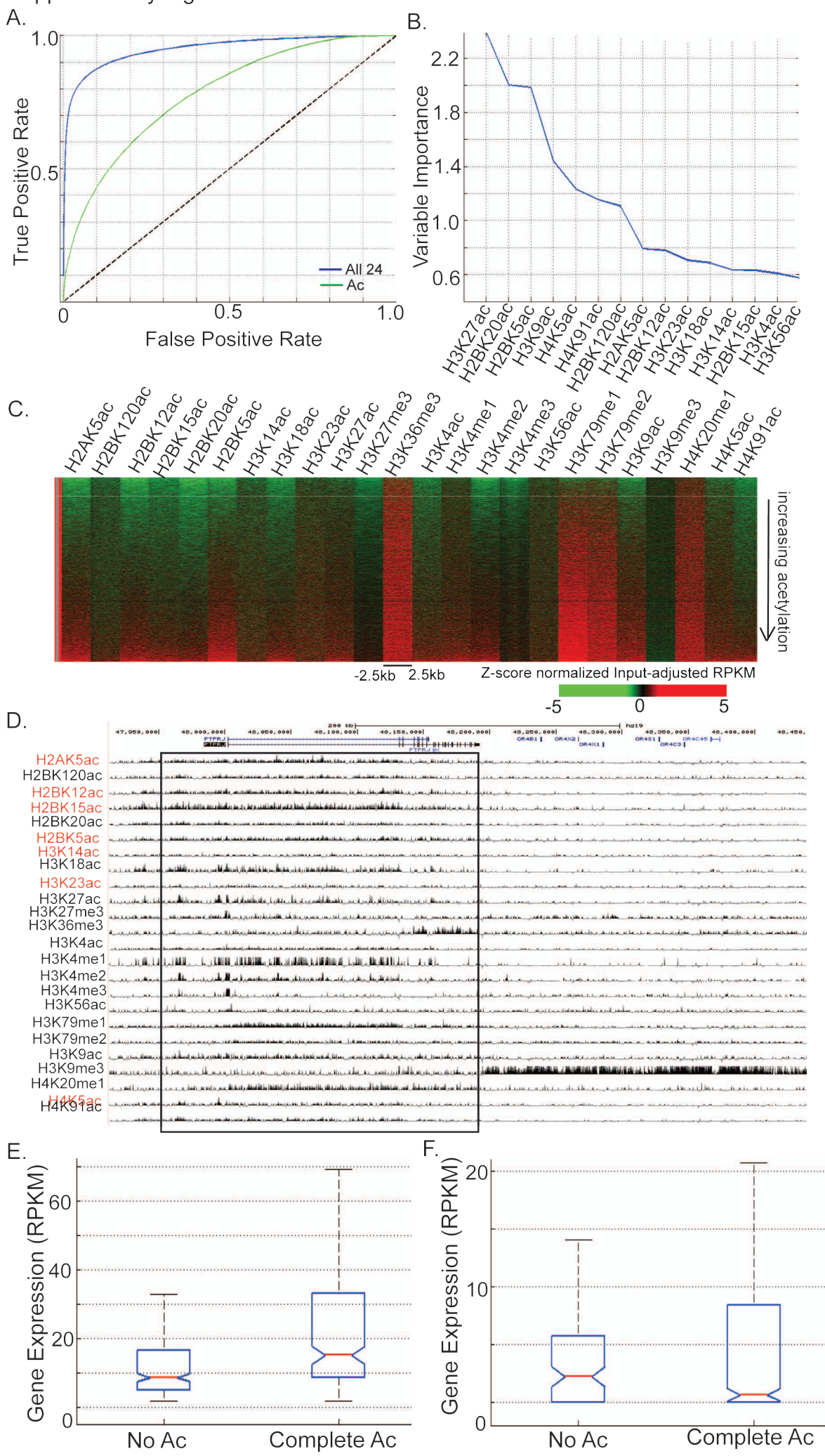

Supplement: Supporting Information [file supp_g3.114.013565_FigureS4.pdf]

Supplementary Figure 5

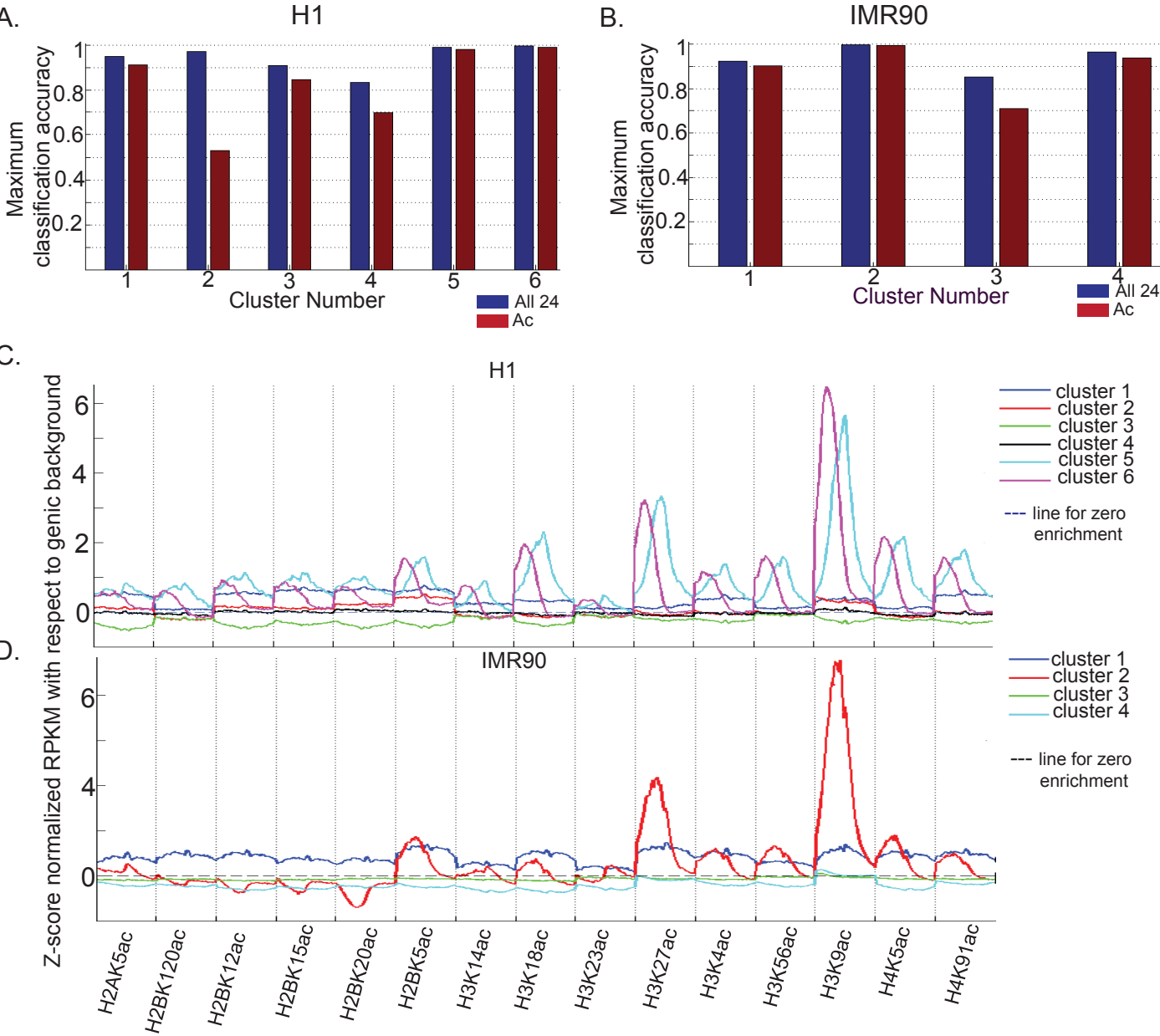

Supplement: Supporting Information [file supp_g3.114.013565_FigureS5.pdf]

Supplementary Figure 6

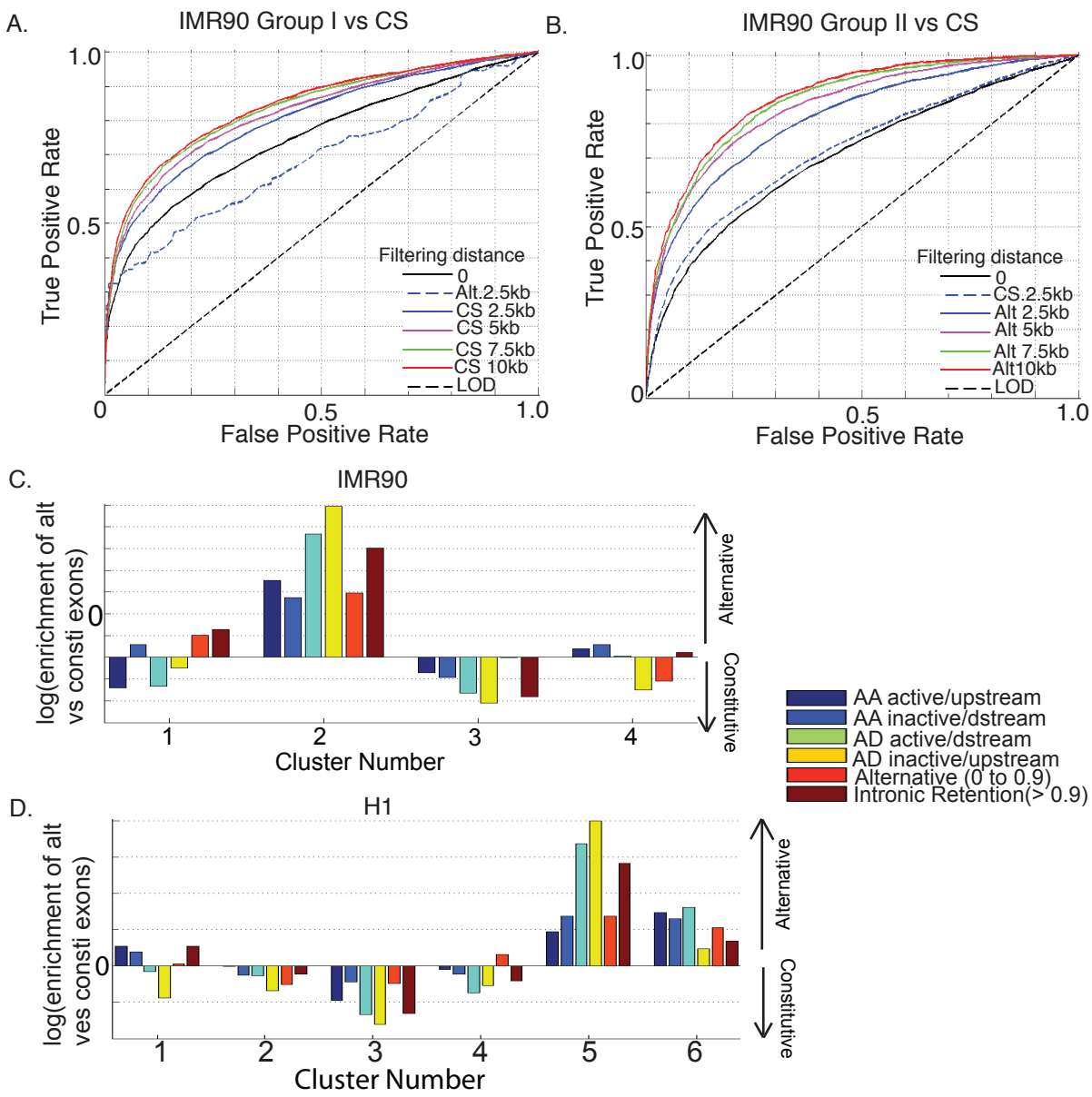

Supplement: Supporting Information [file supp_g3.114.013565_FigureS6.pdf]

Supplementary Figure 7

A.

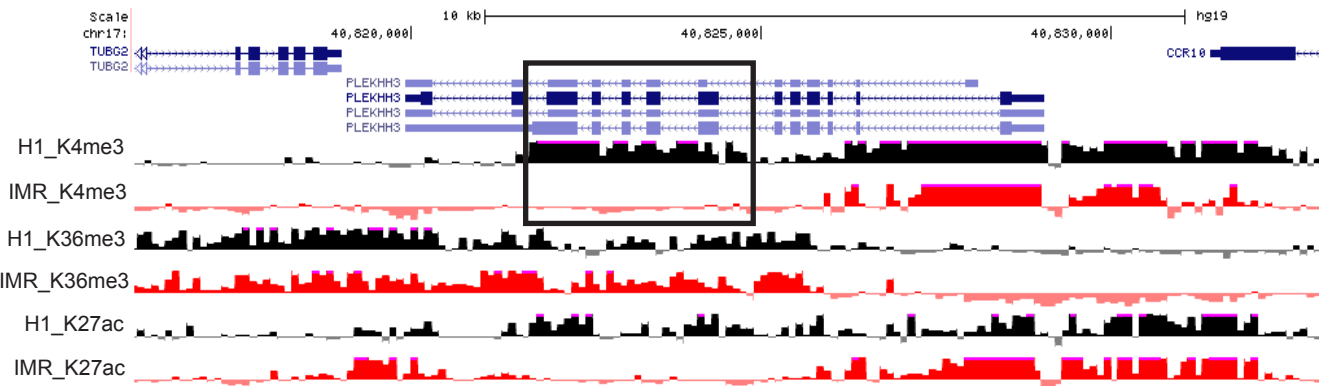

B.

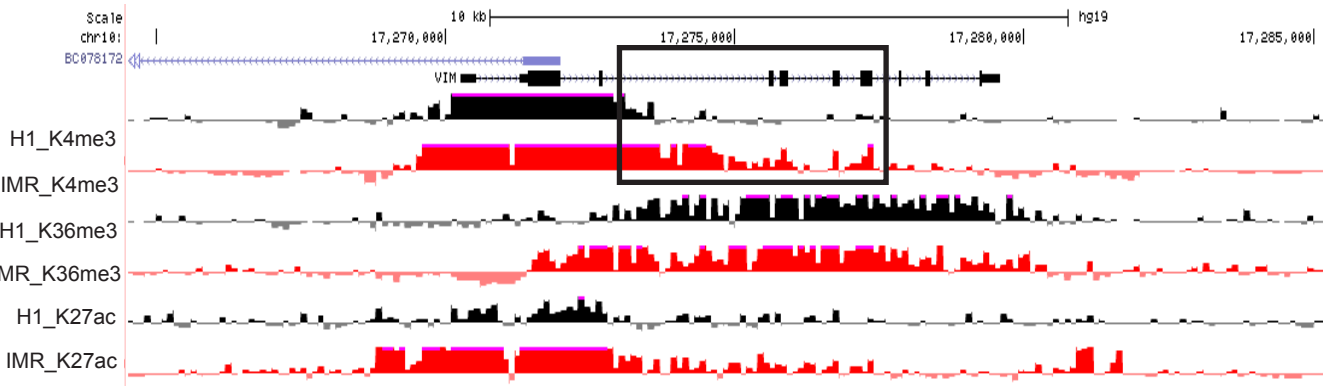

Supplement: Supporting Information [file supp_g3.114.013565_FigureS7.pdf]
